# Guanine quadruplexes are formed by specific regions of human transposable elements

**DOI:** 10.1186/1471-2164-15-1032

**Published:** 2014-11-27

**Authors:** Matej Lexa, Pavlina Steflova, Tomas Martinek, Michaela Vorlickova, Boris Vyskot, Eduard Kejnovsky

**Affiliations:** Faculty of Informatics, Masaryk University Brno, Botanická 68a, 60200 Brno, Czech Republic; Department of Plant Developmental Genetics, Institute of Biophysics, Academy of Sciences of the Czech Republic, Královopolská 135, 61265 Brno, Czech Republic; Department of Computer Systems, Faculty of Information Technology, Božetěchova 1/2, 61266 Brno, Czech Republic; Department of CD Spectroscopy of Nucleic Acids, Institute of Biophysics, Academy of Sciences of the Czech Republic, Královopolská 135, 61265 Brno, Czech Republic; Laboratory of CD Spectroscopy of Nucleic Acids and Proteins, CEITEC - Central European Institute of Technology, Masaryk University, Kamenice 5, 62500 Brno, Czech Republic

**Keywords:** G4 quadruplex, Retrotransposons, Genome

## Abstract

**Background:**

Transposable elements form a significant proportion of eukaryotic genomes. Recently, Lexa et al. (Nucleic Acids Res 42:968-978, 2014) reported that plant long terminal repeat (LTR) retrotransposons often contain potential quadruplex sequences (PQSs) in their LTRs and experimentally confirmed their ability to adopt four-stranded DNA conformations.

**Results:**

Here, we searched for PQSs in human retrotransposons and found that PQSs are specifically localized in the 3’-UTR of LINE-1 elements, in LTRs of HERV elements and are strongly accumulated in specific regions of SVA elements. Circular dichroism spectroscopy confirmed that most PQSs had adopted monomolecular or bimolecular guanine quadruplex structures. Evolutionarily young SVA elements contained more PQSs than older elements and their propensity to form quadruplex DNA was higher. Full-length L1 elements contained more PQSs than truncated elements; the highest proportion of PQSs was found inside transpositionally active L1 elements (PA2 and HS families).

**Conclusions:**

Conservation of quadruplexes at specific positions of transposable elements implies their importance in their life cycle. The increasing quadruplex presence in evolutionarily young LINE-1 and SVA families makes these elements important contributors toward present genome-wide quadruplex distribution.

**Electronic supplementary material:**

The online version of this article (doi:10.1186/1471-2164-15-1032) contains supplementary material, which is available to authorized users.

## Background

Transposable elements (TEs) are abundant inhabitants of eukaryotic genomes, representing e.g. about 50% of the human genome and up to 90% in some plant species. Long terminal repeat (LTR) retrotransposons are most common in plant genomes while animal genomes, including the human genome, are often flooded by non-LTR retrotransposons. Most of the human genome is transcribed and TEs therefore greatly contribute to cellular transcriptome and proteome [1, 2]. Recent insertions of TEs underlie the variability of human populations and can cause several human diseases [3, 4]. Somatic retrotranspositions occur during neuronal development [5, 6] and tumorigenesis [7]. During the last two decades, it became widely accepted that TEs, as an inherently dynamic genome component, have an important role in both cell functioning [8] and genome evolution [9, 10].

Human LTR retrotransposons are represented by endogenous retroviruses (HERV) but their activity is currently very limited: most HERVs were inserted into the genomes of our ancestors earlier that 25 mya [11]. LTR retrotransposons have LTR sequences at both ends, carry GAG and POL genes and several regulatory regions like promoter located inside LTR, primer binding site (PBS) and polypurine (PPT) sites where reverse transcription of the first and second strand of DNA starts, respectively. The majority of human TEs result from the present and past activity of non-LTR retrotransposons, including the LINE-1, Alu and SVA elements [8]. LINE-1 (long interspersed element 1, or L1) have two ORFs coding for RNA binding protein (ORF1) and endonuclease and reverse transcriptase (ORF2). ORFs are flanked with 5’-UTR and 3’-UTR regions. There are at least 850,000 L1 copies in the human genome [12]. Alu elements are about 300 bp long and have dimeric structure formed by the fusion of two monomers derived from 7SL RNA gene. Alus were active over the past 65 mya and the human genome contains more than 1 million copies. SVA elements are about 2 kb long and are composed of a hexamer repeat region, VNTR region, an Alu-like region, a HERV-K10-like region and polyadenylation signal ending with oligo(dA)-rich tail. SVAs were active throughout the last 25 mya of hominoid evolution and have about 3,000 copies [13]. Both Alu and SVA are trans-mobilized by the L1 machinery [14].

Molecular processes participating in the retrotransposon life cycle are regulated both by enzymes encoded by these elements themselves and by several host factors. It is probable that the activity of retrotransposons can also be affected by the changes of DNA conformation that are known to influence many molecular processes (for review see [15]). Formation of multi-stranded DNA structures, namely quadruplex DNA, is probably involved in dimerization of the HIV-1 genomic RNA molecules found in virus particles [16]. Similarly, long polypurine tract (PPT) located in 3’-UTR of L1 retrotransposons, where reverse transcription of the second cDNA strand starts, can form intrastrand quadruplex [17]. Relationship between quadruplexes and transposons can be seen in the cleavage of quadruplexes by RAG1 protein during translocations in human lymphomas [18] because RAG1 protein evolved from transposase of the Transib family of DNA transposons [19].

Recently, we found [20] that potential quadruplex sequences (PQSs) are often located inside LTRs of plant LTR retrotransposons at specific distances from their promoter indicating a possible effect of quadruplexes on transcription. Quadruplexes were better preserved in evolutionary young elements which supports their functional role [20, 21]. Similar observation was made by Savage et al. [22] who found that younger human SVA elements contain more PQS sequences than older SVA elements but the ability of candidate sequences to adopt quadruplex conformation was not experimentally confirmed. Although quadruplexes were found in many regions of human genome, especially in promoters [23–25], systematic analysis of quadruplexes in all main types of human retrotransposons was lacking.

In this study, we searched for PQS sequences in human LINE-1, HERV, SVA and Alu elements. We analyzed the prominent regions of their location as well as the effect of element age and localization on chromosomes. The ability of candidate motifs to adopt quadruplex was verified by circular dichroism and gel electrophoresis.

## Results

### Potential quadruplex-forming sequences are located in specific regions of human transposable elements

We analyzed the localization of PQSs inside main groups of human transposable elements (TEs), namely in LINE-1, Alu elements, HERV retrotransposons and SVA elements. We searched for the (G_*n*_*X*_*n*_*G*_*n*_*X*_*n*_*G*_*n*_*X*_*n*_*G*_*n*_) motif representing potential G-quadruplex cluster inside 894,717 LINE-1 elements, 1,051,161 Alu elements, 38,578 HERV and 5,001 SVA elements or their fragments. Altogether, we found 264,711 PQS in all annotated repeats or their 200 bp flanking sequence (186,507 in plus strands, 78,204 in minus strands). Of those, 183,967 were associated with the four studied classes (136,977 in plus strands, 46,990 in minus strands).

**Figure 1:**
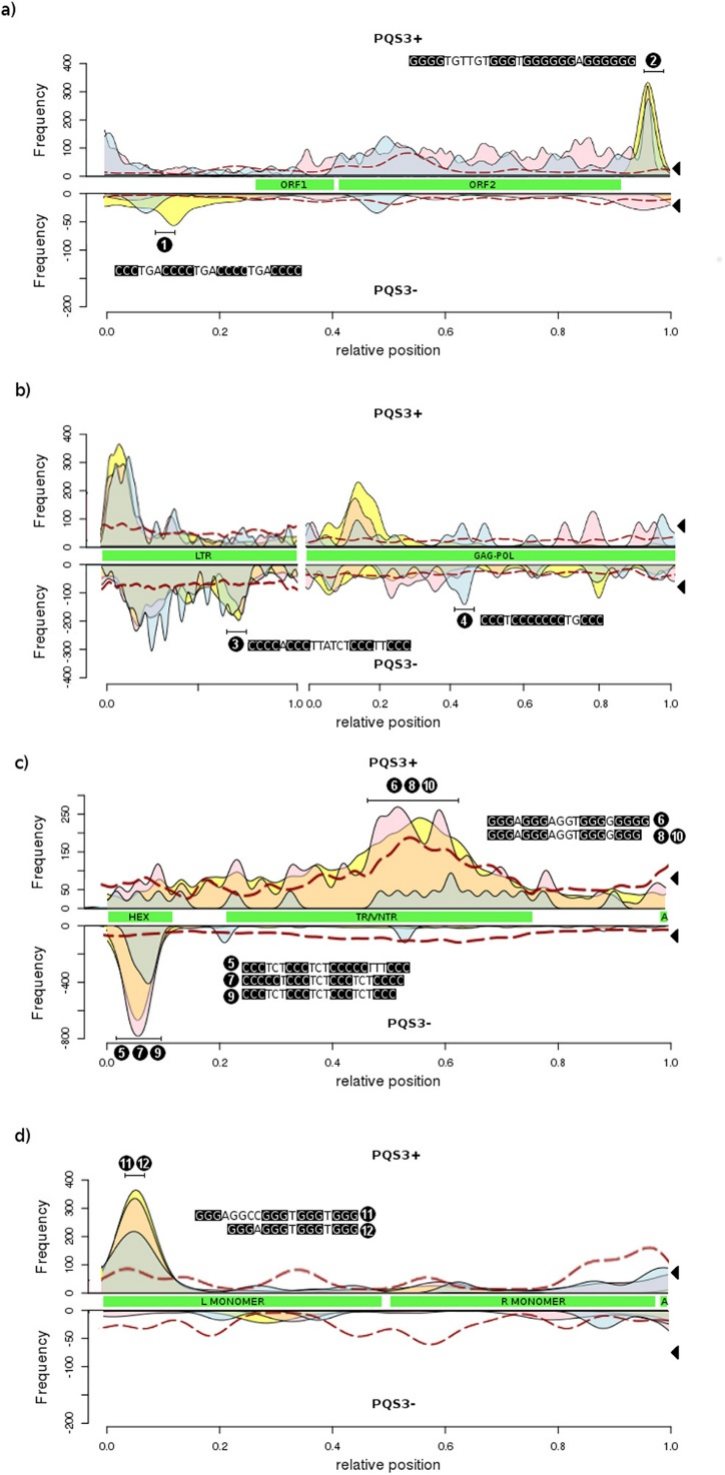
**Occurrence of PQSs along human LINE-1 (a), HERV (b), SVA (c) and Alu (d).** The density of PQS clusters containing a minimum of four adequately spaced GGG groups in the sense strand (PQS3+, upper lines) and antisense strand (PQS3-, lower lines) visualized along LINE-1 **(a)**, HERV **(b)**, SVA **(c)** and Alu elements **(d)**. Sliding window covered between 40-120 bp of the element length. Frequency represents the number of PQSs in such window in the entire family. Green boxes show annotation with main structural components from typical full-length elements (ORF - open reading frame, LTR - long terminal repeat, Hex - hexamer tandem repeat with a CCCTCT consensus, TR/VNTR - SVA tandem repeats with a period of approximately 36, A - polyA tail, L and R MONOMER - 7SL RNA-derived monomer). Chromosomes are visualized separately where autosomes are in yellow, X chromosomes in red and Y chromosomes in blue. The dashed red line shows PQS frequency in randomized control sequences generated by an equivalent 2nd-order Markov chain model. The black triangles on the right show reference densities of PQS sites in the entire human genome recalculated into the coordinates of the given family.

The overall highest abundance of PQSs was observed in SVA (PQS was in 36.2% of elements) followed by LINE-1 (PQS in 7.7% of elements) and HERV elements (PQS in 4.8% of elements). The occurrence of PQSs was lowest in Alu elements (in 1.1% of elements) (a complete list of TE families that contribute more than 1% of PQSs present in the entire genome is available as Additional file 1), showing PQS distribution and frequency in RepeatMasker subfamilies of Alu, ERVL-MaLR, ERVL, ERV1, haT-Charlie, L1, L2, MIR and SVA elements). In LINE-1 elements, PQSs were located almost exclusively in the 3’-UTR region. Only very low numbers of PQSs were found outside this region (Figure 1a). HERV LTR retrotransposons contained PQSs along the whole element with accumulation in LTR regions (Figure 1b). In SVA elements, PQSs were specifically located in Hex region in minus strand and along the larger VNTR region in plus strand (Figure 1c). The occurrence of PQSs inside Alu elements was low throughout most of element length (Figure 1d). There was only one peak of PQS in the left part of left monomer (50 bp from the 5’-end). All mentioned PQS peaks were above the Markov model random background threshold, with SVA-VNTR region being much closer to it than the other PQSs, as expected for a long G-rich tandem repeat.

**Figure 2:**
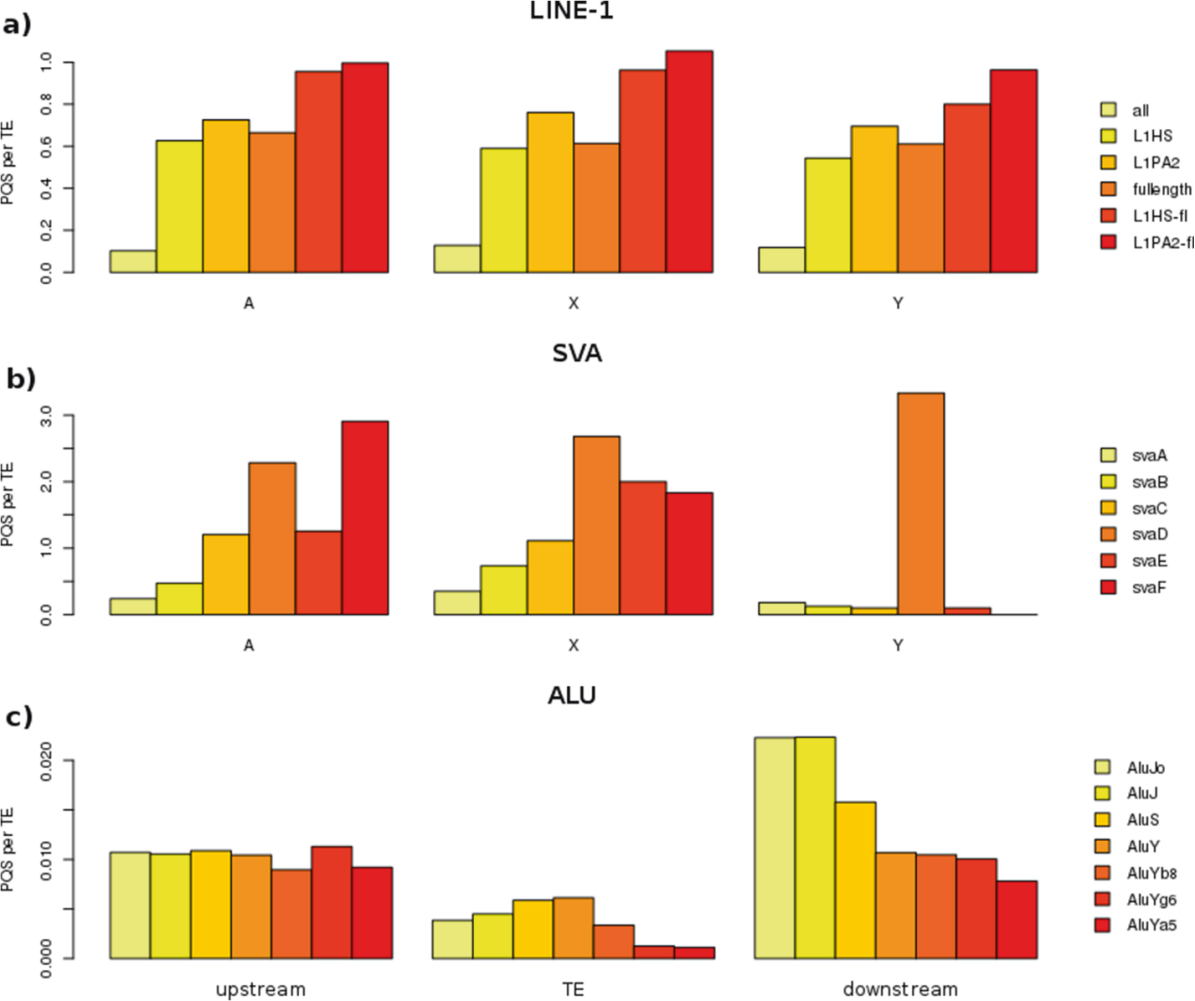
**The abundance of PQSs inside LINE-1, SVA and Alu elements related to the element length, activity or age. (a)** The abundance of PQSs inside all LINE-1, full-length LINEs and active LINEs (L1HS and L1PA2) located on autosomes and X and Y chromosomes. **(b)** The abundance of PQSs inside SVA of different age (oldest SVA-A to youngest SVA-F) located on autosomes and the X and Y chromosomes. **(c)** The abundance of PQSs upstream inside and downstream of Alu elements of different age (oldest Alu-J, middle-aged Alu-S and youngest Alu-Y) located on autosomes and the X and Y chromosomes.

We analyzed the abundance of PQSs in LINE-1, HERV, SVA and Alu elements separately on the Y and X chromosomes and on autosomes. In four main types of elements, the number of quadruplexes was measured on both plus and minus strands for respective chromosomes. The distribution of PQSs along all elements slightly differed between chromosomes being more similar in autosomes and X chromosome and different in the Y chromosome where peaks of PQSs were located in different parts of elements than in autosomes and the X chromosome (Figure 1). The most striking difference between PQS distribution or frequency was observed in the SVA family of transposable elements, where those on the Y chromosome had a reduced PQS content (Figures 1c and 2b). Intriguingly, we noticed an increased occurrence of PQSs in the ORF2 region of LINE-1 elements from chromosome X and Y compared to their autosome counterparts (Figure 1a).

We clustered PQSs from individual families to determine the most common patterns of guanines. We have chosen 2 specific motifs for each TE type among the most common PQS motifs and used them for DNA conformational studies (Table 1). The selected sequences originated from the 5’-UTR and 3’-UTR regions of LINE-1, LTR and gag-pol gene of HERV, Hex and VNTR regions of SVA and left part of the left monomer of Alu (Figure 1a-d).

**Table 1:** **Oligonucleotides used in this study**

Number	Name	Sequence	Length [nt]
1	L1_1	TAGGTGCTC **GGGG** TCA **GGGG** TCA **GGGG** TCA **GGG** ACCCACTTG	42
2	L1_2	ATCACACTCT **GGGG** TGTTGT **GGG** T **GGGGGG** A **GGGGGG** AGGATAGCATT **GGG** AGATATACC	60
3	HERV_1	AAAGAGTCA **GGG** AA **GGG** AGATAA **GGG** T **GGGG** CCGTTTTAT	40
4	HERV_2	TAAATTGCT **GGG** CA **GGGGGGG** A **GGG** CTAGTCACG	34
5	SVA-A_HEX	GGAGATCAA **GGG** AAA **GGGGG** AGA **GGG** AGA **GGG** AGAGGCCAA	41
6	SVA-CF_VNTR	CGCCCGTCC **GGG** A **GGG** AGGT **GGGGGGGG** TCAGCCCCC	37
7	SVA-C_HEX	GGAGACCGT **GGGG** AGA **GGG** AGA **GGG** A **GGGGG** AGAGGAGAC	40
8	SVA-BF_VNTR	GCCCCGTCC **GGG** A **GGG** AGGT **GGGGGGG** TCAGCCCCC	36
9	SVA-F_HEX	GGAGAGAGA **GGG** AGA **GGG** AGA **GGG** AGA **GGG** AGA **GGG** AGAGTGCTG	45
10	SVA-F_VNTR	GTGCCATCC **GGG** A **GGG** AGGT **GGGGGGG** TCAGCCCCC	36
11	ALU-S_1	CCAGCACTTT **GGG** AGGCC **GGG** T **GGG** T **GGG** TCACCTGAGG	39
12	ALU-S_2	CCAGCACTTT **GGG** A **GGG** T **GGG** T **GGG** TGGATCACTT	35

Names and the sequences of oligonucleotides are shown. Clusters of three or more guanines are shown as bold.

### The abundance of PQSs in the neighborhood of transposable elements

**Figure 3:**
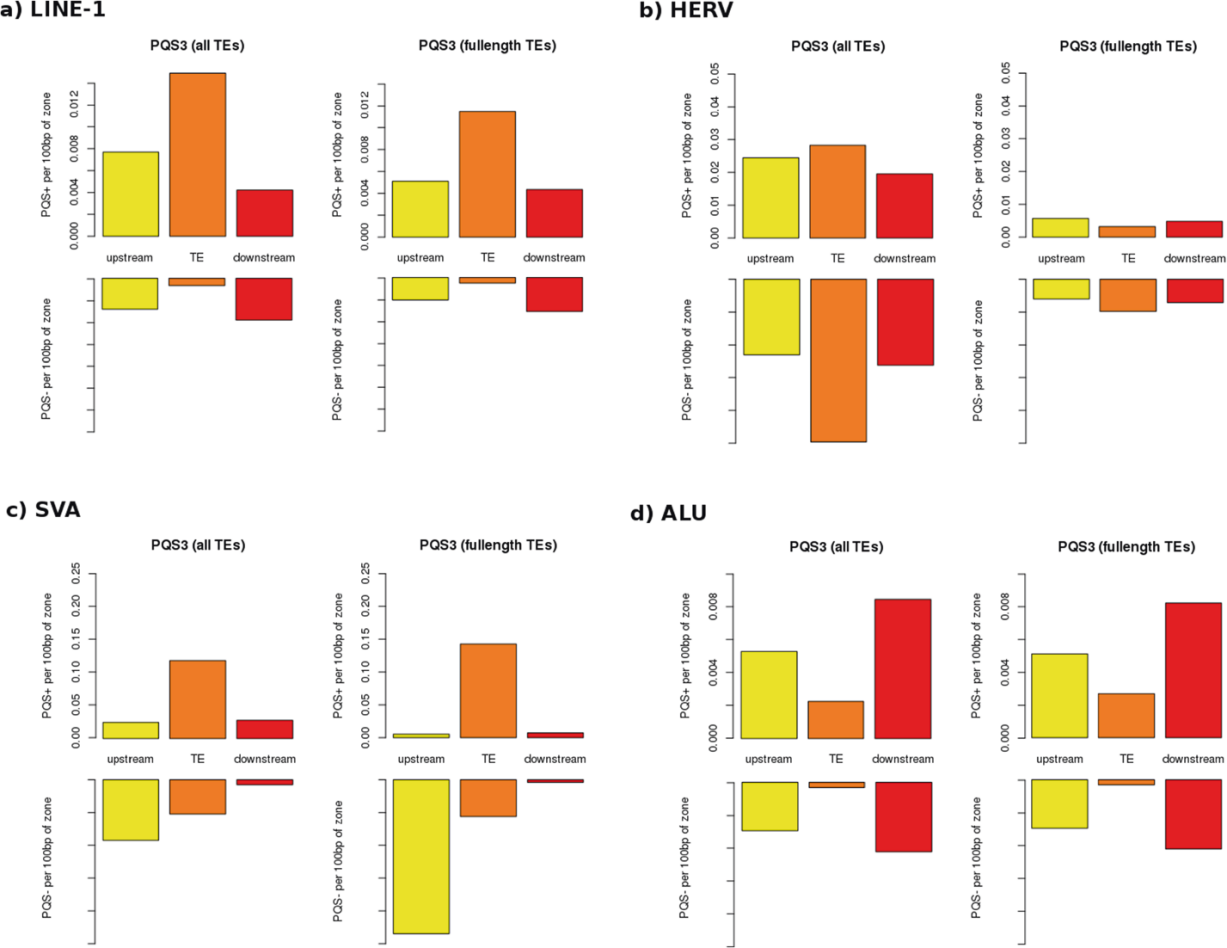
**The density of PQSs inside TEs and in TEs vicinity.** The number of PQSs per 100 bp of zone located inside, upstream and downstream of LINE-1 **(a)**, HERV **(b)**, SVA **(c)** and Alu elements **(d)**. For each element type PQS in plus strand (upper row) and minus strand (lower row) are shown. We analyzed either all elements (left figures) or only full-length elements (right figures).

We compared the abundance of PQSs inside and in the vicinity of LINE-1, HERV, SVA and Alu elements. In full-length LINE-1 elements, the density of PQSs was markedly higher inside elements than in element vicinity. The greater abundance of PQSs inside LINE-1 elements compared to the element vicinity was observed only in plus strand while the neighborhood contained more PQSs than element when minus strand was analyzed (Figure 3a). Elements with the 3’-UTR PQS were much less likely to have the PQSs in the 3’ downstream flanking region (data not shown). In HERV elements, many more PQSs were present inside elements than in their neighborhood, especially when full-length elements were taken into account (Figure 3b). High enrichment of elements compared to their neighborhood was also observed in SVA elements (Figure 3c). This trend was stronger in plus than in minus strand. In minus strand, SVA contained more PQSs upstream than downstream of elements. Alu elements differed from LINE-1, HERV and SVA. The density of PQSs inside Alu elements was lower than in regions located upstream and downstream (Figure 3d).

### The abundance of PQSs within transposable elements of different age and activity

We compared the PQS abundance in all LINE-1 elements, full-length LINE-1 and transcriptionally active LINE-1 families (L1HS and L1PA) [26]. We found that full-length LINEs contained much more PQSs than truncated LINE elements (Figure 2a). Among full-length elements, the transcriptionally active L1HS and L1PA2 families contained more PQSs than was the average abundance of PQSs inside full-length LINEs. Truncated L1HS and L1PA2 homologues contained much less PQSs. These trends were observed both on autosomes and on X and Y sex chromosomes.

We analyzed the abundance of PQSs inside SVA elements of different age - SVA-A (oldest family) to SVA-F (youngest family). We found that the abundance of PQSs was higher in younger elements (SVA-D, SVA-E and SVA-F) than in older elements (SVA-A, SVA-B and SVA-C) and this trend was same both in autosomes and sex chromosomes (Figure 2b). The abundance of PQSs was highest in middle-aged SVA elements (Figure 2b). The PQSs were common in the central part of elements in plus strand. Detailed analysis revealed that in older elements, the PQS abundance in the central part of plus strand decreased and predominated in the left part of SVA in the minus strand (not shown). The peak of PQSs in SVA-E present on the Y chromosome was caused by the low number of elements and the SVA-F elements even absented on the Y chromosome.

We made similar analysis of Alu elements where Alu-J are oldest, Alu-S are middle-aged and Alu-Y are youngest elements. We found that in contrast to LINE-1 and SVA, the age did not markedly affect the abundance of PQSs inside Alu elements. There is a slight PQS-increasing trend with age in the main families, however the youngest subfamilies (AluYg6, Ya5) [27] are also depleted of PQSs (Figure 2c). Because Alu elements have more PQSs in their vicinity than inside elements (Figure 3) we also analyzed the upstream and downstream regions. We found that older Alu elements contained more PQSs than younger elements in their downstream regions (Figure 2c). The PQS abundance did not differ markedly between autosomes and sex chromosomes, a small decrease in PQSs on the Y chromosome was registered (Figure 1d). The most active families of Alu (AluYg6 and AluYa5) had lower abundance of PQSs than average Alu-Y elements.

### PQSs can form quadruplexes as revealed by circular dichroism

We probed DNA conformational properties of 12 oligonucleotides (Table 1) representing PQSs obtained from SVA, HERV, LINE-1 and Alu elements by circular dichroism (CD). We tested their ability to form quadruplex structures upon increasing concentration of potassium ions.

**Figure 4:**
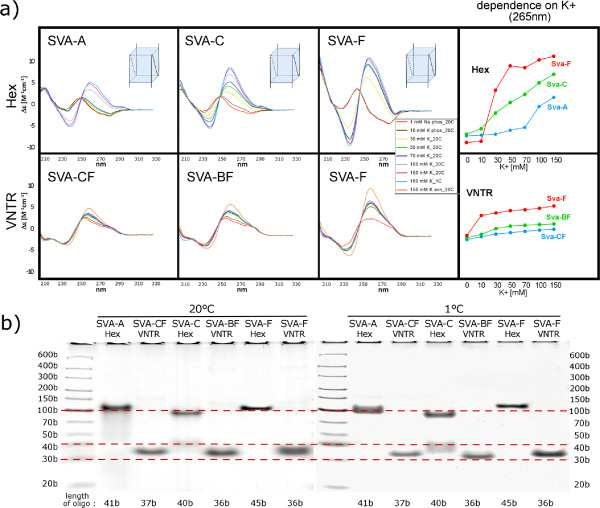
**Circular dichroism measurements and gel electrophoreses of PQSs from SVA elements of different age. (a)** CD spectra of SVA-A, SVA-C and SVA-F (shown in Table 1) obtained at various concentrations of potassium ions and at various temperatures are marked by different colors. The peak at 265 nm corresponds to a parallel-stranded quadruplex. Sketches correspond to the most probable folding of the dominating quadruplex structure according to CD and electrophoretic results. **(b)** Native gel electrophoreses of SVA PQS in the presence of 150 mM KCl at 20°C or 1°C.

First, we measured CD spectra of PQSs originating from SVA elements because they contain PQSs more often than any other human TEs. We divided SVA elements into three families with different age - oldest SVA-A family, middle-aged SVA-C family and youngest SVA-F family - and for each family we analyzed the ability of one Hex region and one VNTR region to adopt a quadruplex structure. VNTR consensus sequences in older families were always present in younger families as well, therefore we used consensus sequences common for multiple families - SVA-BF for families B to F and SVA-CF for families C to F. Only SVA-F VNTR oligonucleotide was specific for the youngest SVA family. As shown in Figure 4a, the positive CD band at about 260 nm, which is characteristic of the presence of a parallel quadruplex [28], increased steeply and at lower potassium concentrations with SVA-F (youngest) than with SVA-C (Figure 4a). Much less increase in this band and only at the highest K^+^ concentrations used was observed with the older SVA-A family and the common consensus oligonucleotides SVA-BF and SVA-CF. Similarly, the thermal stability of quadruplexes was highest in SVA-F and lowest in SVA-A (not shown). Native gel electrophoresis at 150 mM K^+^ showed that Hex region of three groups of SVA adopted bimolecular quadruplexes (Figure 4b). VNTR region provided CD spectra of the B-DNA type at low K^+^ concentrations marked out by low amplitudes and a slightly predominating 260 nm band, which is characteristic of duplexes of G-rich and C-rich DNA strands [29]. These monomolecular structures (Figure 4b) may thus correspond to hairpins containing rather accidental, namely G.C, base pairs. The increase in the 260 nm band with increasing K^+^ concentration indicating quadruplex formation was again most obvious with SVA-F and less so with SVA-BF and SVA-CF. The quadruplexes were formed non-cooperatively and not much willingly.

**Figure 5:**
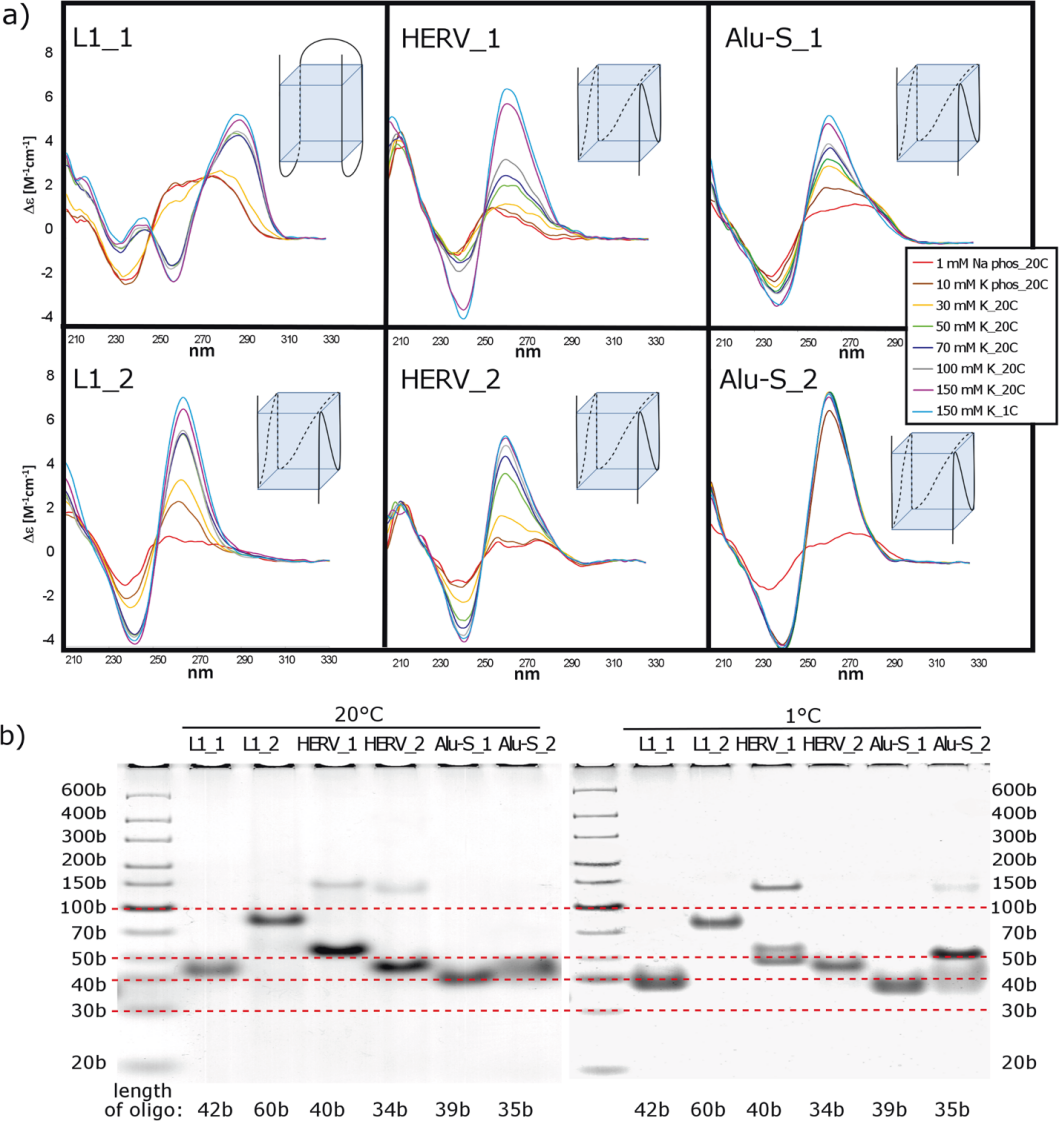
**Circular dichroism measurements and native gel electrophoreses of PQSs from LINE-1, HERV and Alu elements. (a)** CD spectra of the oligonucleotides (shown in Table S1) obtained at various concentrations of potassium and at various temperatures are marked by different colors. The peak at 265 nm indicates formation of the parallel-stranded quadruplex while maximum at 295 nm corresponds to an antiparallel-stranded quadruplex. Sketches correspond to the most probable folding of the dominating quadruplex structure according to CD and electrophoretic results. **(b)** Native gel electrophoreses of LINE-1, HERV and Alu PQS in the presence of 150 mM KCl at 20°C or 1°C.

PQSs originating from the LINE-1 elements were selected from the 5’-UTR and 3’-UTR regions (Figure 1). The PQSs from the 5’-UTR (labelled as L1_1) provided CD spectrum corresponding to antiparallel quadruplex (Figure 5a), while the CD spectrum of the PQS from 3’-UTR (labelled as L1_2) corresponded to that of the parallel-stranded quadruplex (Figure 5a). Native PAGE revealed that both the anti-parallel L1_1 and the parallel L1_2 quadruplexes were monomolecular at low as well as at room temperature (Figure 5b). Antiparallel folding of the L1_1 quadruplex was enabled by the sufficiently long (trinucleotide) loops between all four G blocks.

Two PQSs were selected from HERV elements. The first PQS corresponded to a minor PQS peak in the LTR in the minus strand (HERV_1) and the second PQS originated from the gag-pol region of the minus strand (HERV_2, Figure 1). CD measurements indicated gradual formation of parallel-stranded quadruplexes with both PQSs (Figure 5a). Native PAGE revealed that a monomolecular quadruplex structure dominated in both, HERV_1 and HERV_2 at room temperature, while a bimolecular quadruplex, in addition to two types of monomolecular ones, were formed by HERV_1 at low temperatures (Figure 5b).

In Alu elements, two PQSs (Alu-S_1 and Alu-S_2) were selected for CD measurements, both from the left part of left monomer located in plus strand (Figure 1). Both PQSs corresponded to the middle-aged Alu elements (Alu-S). Although CD spectra of both oligonucleotides indicated the formation of parallel-stranded quadruplex, the spectral changes induced by the increasing potassium concentration were gradual and limited in the case of Alu-S_1. This along with the shoulder on the long wavelength part of the positive 260nm CD band (B-DNA displays a positive maximum around 280nm) indicates that a substantial part of Alu-S_1 sequence formed a hairpin. Alu-S_2 formed the quadruplex at much lower potassium concentration (Figure 5a) and the transition was highly cooperative. The quadruplex was parallel and intramolecular in the same way as was the quadruplex of Alu-S_1 (Figure 5b). Note that the mobility of the studied quadruplexes is slower than would correspond to their length markers. This is usually the case with the heavy G-rich strands. Moreover, the mobilities of the intramolecular qudruplexes differ (more than follows from their lengths), which may be partly a consequence of their distinct compactness, and mainly, by distinct hindering effects of the overlapping nucleotides not involved in the quadruplex structure. In addition, we measured PQSs selected from older and younger Alu families (Alu-J and Alu-Y, respectively) but we found no correlation between susceptibility to form quadruplex and the age of Alu elements.

## Discussion

We found that potential quadruplex-forming sequences are located in specific regions of human transposable elements and experimentally verified the ability of such sequences to adopt quadruplex DNA conformation. Full-length and active L1 elements and younger SVA elements had a larger number of PQSs. The propensity of these sequences to form quadruplex and quadruplex stability (not shown) were higher than in older elements. Alu elements contained PQSs not inside but in their neighborhood where more PQSs were present in downstream regions of older elements.

Two available counts of G4-quadruplexes in the entire human genome found about 375,000 PQSs [24, 30]. This allows us to express our numbers as proportions of mobile element PQSs to whole-genome PQS content with a value of 71%. The four main classes of elements studied here carry 49% of total predicted PQSs. These numbers reflect the current human genome sequencing and annotation status and are very likely to miss potential PQSs in centromeres, telomeres or other difficult-to-map regions of the human genome.

Our results are in agreement with Savage et al. [22] who also found that the youngest SVA (SVA-E, SVA-F) contained more quadruplexes than older elements. Such age-dependent distribution of PQSs (Figure 2) can be explained by the action of constraints leading to fixation of quadruplexes in recent and active elements while non-active older elements accumulate mutations that hinder quadruplex formation. Moreover, we found that quadruplexes are present in the central part of SVA elements in plus strand and in the left part of minus strand. If the localization of quadruplexes in plus strand has negative effect on transcription and their presence in minus strand has a positive effect [15, 21], then the potential evolutionary balancing of quadruplexes abundance (an increase or a decrease) in complementary strands could regulate element activity over time.

The greater abundance of PQSs (that are GC-rich) in the neighborhood of older Alu elements is probably related to generally high GC-content of isochores containing older Alus [31]. Surprisingly, despite the age-dependent increase of GC-content of Alu neighborhood, the abundance of PQSs inside Alu elements was very low (Figure 3) and did not increase with the element age (Figure 2).

We have shown that PQSs are strongly accumulated in 3’-UTR of LINE-1 elements. Quadruplexes located in 3’-UTR can have an effect on target-primed reverse transcription (TPRT) that starts at the 3’ end. Quadruplexes formed either by RNA template or by the growing first DNA strand can represent a barrier for reverse transcription. However, quadruplex DNA can regulate not only the transposable element itself but can also influence neighboring genes as was proposed recently by Kejnovsky and Lexa [21]. Because SVA elements are preferentially located inside genes or in their neighborhood [22] we suggest that recent SVA elements could spread quadruplex motifs close to genes or into genes and in this way they regulate expression of these genes. The regulatory potential of quadruplexes inside TEs decreases as the element gets older and is eroded by mutations and rearrangements. In this way, quadruplexes can enlarge the potential of transposable elements to respond to environmental challenges as was suggested by McClintock [32] long time ago.

Quadruplexes carried by TEs can also affect other cellular processes like replication or epigenetic regulation. It is remarkable that quadruplexes are located close to the LINE-1 poly(dA) tail that represents the labile region of duplex DNA. Other labile (AT-rich) regions are represented by replication origins and, surprisingly, also here quadruplexes are located [33]. Because quadruplexes also represent barriers for replication, or at least can slow it down, the spreading of PQSs by retrotransposons can also contribute to the regulation of replication speed. In addition, the quadruplexes can represent epigenetic marks in large introns that contain repetitive DNA and are also AT-rich [21, 34]. Moreover, if non-B DNA conformations are nucleosome-free [35, 36] and some transposable elements are preferentially inserted into naked DNA [37], then one would expect that such regions could represent sites for nested insertions, at least in some TE families.

Several proteins were shown to bind quadruplex DNA [15, 38]. For example, p53 protein, that has binding sites inside human Alu and L1 elements [39, 40], can strongly bind quadruplex DNA [41]. Another example is the recombination and repair protein Ku70 that was shown to bind cDNA of Ty1 yeast retrotransposons [42] and has high affinity to quadruplex DNA [43]. In this context, it is interesting that human LINEs have many Ku70/80 binding sites [44].

Taken together, the remarkable ability of some proteins to bind both TEs and quadruplex DNA underlining the relationship of these unusual DNA conformations with transposable elements as well as the higher abundance of PQSs inside younger, full-length and active elements indicates the role of quadruplexes in TE spreading. Such a role can consist in negative or positive regulation of TE activity, e.g. in response to current intracellular ionic conditions influencing the stability of quadruplexes. In the long-term perspective, quadruplexes can represent an evolutionary feedback suppressing non-controlled amplification of active elements.

## Conclusions

The results suggest that activity of transposable elements, especially LINE-1 and SVA elements, contributes towards genome-wide quadruplex distribution in human. Conservation of quadruplexes at specific positions implies their function either in the life cycle of transposable elements or host genome maintenance, or both. All tested PQSs were able to form quadruplex structure in vitro, albeit with differing willingness, strand orientation and molecularity. LINE-1 and SVA families displayed an age-dependent pattern with younger elements containing a higher number of more stable quadruplexes. Further studies should be done to determine how the conserved elements are selected for during evolution.

## Methods

### Search for potential quadruplex-forming sequences inside transposable element

Repetitive sequences in the human genome were collected using UCSC Table Browser data [45]. The repeats from Repeat Masker track [46] (RepeatMasker, www.repeatmasker.org) from the hg38 version of the human genome were extended 200 bp in both directions and exported from Table Browser in FASTA format. The header of each sequence contained the precise position of each sequence in the hg38 assembly of the human genome, including the harboring chromosome. It also identified the class and family of element by name as returned by Repeat Masker. These identifiers were used in assigning data and results to repeats, chromosomes or to calculate whether a detected feature was inside or outside the studied repetitive region. A feature was considered to be inside only if one of its ends localized to the TE proper (not the flanking region). This dataset also includes truncated or fragmented sequences. In selected analyses, we used full-length elements, using only TEs that were longer than two thirds of a typical representative, resulting in the following thresholds [given in bp]: L1 - 4,700, Alu - 250, SVA - 1,600, HERV (ltr) - 300, HERV (internal) - 2000.

The collected sequences were scanned for the occurrence of the typical PQS3 pattern GGG-N_1-7_-GGG-N_1-7_-GGG-_1-7_-GGG on both strands and labelled PQS3+ and PQS3-, respectively. The scan used a Perl script based on the regular expressions used in our previous study [20], recording the position and identity of each PQS3 pattern for subsequent counting and plotting. To verify that PQS frequency is not simply determined by the overall GC-content of the respective region, we calculated the expected number of PQSs in a random sequence generated by a second-order Markov model. This model was derived from the original sequence in windows of 150 bp as described previously [20, 23].

### CD spectroscopy and polyacrylamide gel electrophoresis

High-quality oligonucleotides (lyophilized) were purchased from Generi Biotech (Hradec Králové, Czech Republic) and dissolved in 1 mM sodium phosphate buffer with 0.3 mM EDTA (pH 7.0) to obtain final stock concentration 100 OD.ml^-1^. Chemicals of analytical grade (Sigma-Aldrich) and deionized water (18 × 10^6^ ohm resistance, Elga) were used for buffers. The exact oligonucleotide concentration was determined by absorbance measurements of appropriately diluted samples at 90°C in the above buffer using Unicam 5625 UV/VIS spectrophotometer and molar extinction coefficients calculated according to Gray et al. [47]. Before any measurements the DNA samples were denatured for 2 min at 90°C and slowly cooled to room temperature.

CD measurements were done using a Jasco 815 dichrograph in 1 cm Hellma cells, placed in a temperature-controlled holder. Circular dichroism was expressed as the difference in the molar absorption of the left-handed and right-handed circularly polarized light, *Δε* in units of M^-1^*cm*^-1^. The molarities (M) were related to nucleosides. Experimental conditions were changed directly in the cells by adding concentrated solutions of potassium chloride and the final sample concentration was corrected for the volume increase. All the presented K^+^ dependences were measured at 20° and 1°C.

Native polyacrylamide gel electrophoresis was performed in a temperature-controlled electrophoretic apparatus (SE-600; HoeferScientific). The gel concentration was 16% (29:1 monomer to bis ratio; Applichem). Two micrograms of oligonucleotide dissolved in 10 mM potassium phosphate and 135 mM potassium chloride were loaded into each lane. Samples were electrophoresed in 70 mM concentration of K^+^ ions at 20°C for 18 h at 30V or at 1°C for 18 h at 55V. Gels were stained with Stains All (Sigma) after electrophoresis and scanned using the Personal Densitometer SI, model 375-A (Molecular Dynamics).

## Electronic supplementary material

Additional file 1: **A detailed visualization of PQS coverage of main human transposable element families and subfamilies.** (PDF 4 MB)
