# Supplementary material for: Guanine quadruplexes are formed by specific regions of human transposable elements
Source: BMC Genomics. 2014 Nov 27;15(1):1032. doi: 10.1186/1471-2164-15-1032 (PMC4407331; doi:10.1186/1471-2164-15-1032)

Supplementary data

This supplementary data contains diagrams showing the detailed analysis of potential quadruplexes (PQS) in selected repeat families, namely: Alu, ERVL-MaLR, ERVL, ERV1, haT-Charlie, L1, L2, MIR and SVA elements. Each diagram shows information about all subfamilies of the given repeat family (sorted by amount of PQS in descending order). The diagram is composed of three major parts:

1) **Repeat statistics** section includes following columns:
   a) **Name:** Represents the subfamily name.
   b) **Avg. len.** and **Max. len:** Represent the average and maximum repeat length (it means the whole repeat, not just the fragment). These items are calculated from lengths assigned to repeat by RepeatMasker program.
   c) **F5 cov., Max. cov.** and **F3 cov.**: Represent the coverage for 5' flank region, repeat body and 3' flank region. The coverage generally represents the number of elements stacked above themselves at a given genome position. As the repeats are usually spread along the genome in form of fragments, it is not possible to calculate the overall number of repeats (we do not know easily which fragments belong to each other). Instead the coverage shows how much a given part of the repeat is included in a genome. Then **Max. cov.** represents the maximal coverage along the whole repeat, while **F5 cov.** and **F3 cov.** correspond to the number of detected flank regions. Please note that all these three numbers differ, because 5' and 3' flank regions are not assigned for each repeat body (see section Methodology for detailed information). The length of flank regions was set to 500 bp.
2) **Quadruplexes count** section includes columns: **F5 (+/-)**, **Rep. (+/-)** and **F3 (+/-)** , that represent the number of PQS in repeat 5' flank region, body and 3' flank region. The sign (+) means the number of PQS detected on the same strand as the repeat, while the sign (–) corresponds to the numbers on the opposite strand.
3) **Repeat Map** section represents the three heatmaps that highlight PQS positions in 5' flank region, repeat body and 3' flank region. Each heatmap is further composed of three color stripes. The middle one represents the coverage of the repeat such that you can see which parts of the repeat are often included in the genome. As shown in the legend below, the colors are scaled to range between zero (white color) and one (red color), where the zero means that the given part of the repeat is not included in the genome while the one means that the occurrence of repeat fragment corresponds to the maximum coverage (**Max. cov.** item described above). Top and bottom stripe represents the occurrence of PQS in + and - strand, where + strand meas the same strand as the repeat and – strand represents the opposite one. Colors in top and bottom stripe are also scaled to range between zero and one. In this case, zero means no occurrence of PQS, while a value of one corresponds to the maximal number of PQS in the given repeat or flank region (**F5 (+/-)**, **Rep. (+/-)** and **F3 (+/-)** items described above). Please note, that the selected color scheme shows relative number of PQS in the given subfamily and therefore some PQS are shown in red color just because they are the only ones in the given subfamily. On the other hand, had we chosen absolute color scheme across the whole family, we would not see some interesting details in low-coverage subfamilies.

Methodology

This section describes in detail the methodology of obtaining the diagrams listed in this supplementary data. The procedure differed in minor points from the one used in the main paper, namely using the hg19 version of the human genome, including a few extra data cleaning actions or working with longer flanking regions. It included the following steps:

1. **Detection of PQS:** Potential quadruplexes were detected using the regular expression following the usual quadruplex formula $G_{3+}N_{(1-7)}G_{3+}N_{(1-7)}G_{3+}N_{(1-7)}G_{3+}$, where N represents the loop between groups of guanines. As the groups of possible guanines and loops can continue and form a possible chain of PQS we extended the regular expression to capture all sequences capable to form PQS at one or more positions.
2. **Detection of repeats:** The positions of individual repeats was obtained using the RepeatMasker program. We used precalculated data in configuration: hg19 - Feb 2009, RepeatMasker (RMSK) open-4.0.3 - Repeat Library 20130422, available at webpage: http://www.repeatmasker.org/species/hg.html. Parts of RMSK output had to be corrected or filtered, because RMSK printed repeat fragments with wrong lengths, e.g. some of them had length 1 bp etc.
3. **Calculation of repeat flank regions:** For analysis of PQS in proximity of repeats, it is necessary to link individual repeats with their flank regions. However, for detection of repeat flank regions a several problems have to be solved: 1) Repeats are usually available in form of fragments and we do not know which ones belong to each other. 2) Some of available repeat fragments are too distant from their potential start or end positions and thus the prediction of the appropriate flank regions would be inaccurate. To solve these problems we used two heuristics. In the first case we grouped fragments of the potential repeat only if: (a) they were placed on the same strand, (b) RMSK positions (RepStart, RepEnd, RepLeft) continues in ascending order along the repeat and (c) the distance between consecutive fragments does not exceed 10.000 bp. The second problem was solved as follows: the position of potential flank regions was calculated from potential start and end repeat position only if the distance between the first/last repeat fragment and the potential start/end position does not exceed 10% of whole repeat length. We assume that the proportion according to repeat length will work for short and long repeats better than the constant distance. Also, we are aware of possible discussion about individual parameters setting. All parameters are configurable and selected values seemed to give the reasonable results.
4. **Filtration of flank regions:** As the eukaryotic genomes are rich in repeats, it is not unusual that flank region of single repeat overlaps with fragments of another repeat. For this reason we filtered all flank region overlapped with any other repeat, except of simple repeats or low complexity regions.
5. **Calculation of PQS overlapped with repeat or flank regions:** In the next step, we calculated the overlaps between PQS and repeats and their flank regions. More specifically, PQS was considered to be a part of the repeat or its flank region only if they overlapped in at least 10 bp (this constant was selected based on distribution of PQS lengths and it should cover more than half of quadruplex length for 80% of all PQS).
6. **Generation of diagrams:** Finally, the positions of overlapped regions were recalculated according to repeat or flank start position. Repeat statistics, PQS count and repeat heatmaps were printed in the form of diagram.

All listed steps were implemented in R language using basic Bioconductor packages. Source codes and calculated data are available online at webpage:
http://www.fit.vutbr.cz/~martinto/pubs/pqs_hg19.zip

Alu (1/2)

Repeat Statistics / Quadruplex Count

Name	Avg.len.	Max.len.	F5 cov.	Max.cov.	F3 cov.	F5 (+/-)	Rep. (+/-)	F3 (+/-)
AluJb	312	320	104,137	117,190	109,551	1,947 / 1,674	405 / 148	3,195 / 2,088
AluJr	312	320	60,782	65,989	57,616	1,382 / 1,127	180 / 94	1,809 / 1,241
AluSx1	312	312	101,930	108,566	103,019	2,304 / 1,972	175 / 48	2,728 / 2,270
AluJo	312	320	54,695	57,968	52,800	1,253 / 1,072	141 / 80	1,736 / 1,104
AluSx	312	320	113,072	123,248	117,889	2,341 / 2,096	186 / 30	3,049 / 2,338
AluSz	312	312	94,370	96,757	87,610	1,903 / 1,775	142 / 39	2,157 / 1,796
AluY	311	320	109,624	115,522	113,313	1,809 / 1,413	81 / 6	1,974 / 1,759
AluSq2	312	312	50,330	53,976	52,657	935 / 839	71 / 13	1,210 / 876
AluSz6	312	312	38,021	42,379	40,934	648 / 552	63 / 15	889 / 661
AluSx3	312	320	24,424	27,695	26,173	397 / 335	59 / 14	493 / 432
AluJr4	312	320	13,523	15,119	13,515	213 / 174	39 / 29	320 / 196
AluSg	310	310	34,486	35,336	33,831	582 / 522	61 / 4	701 / 559
AluSp	313	313	45,532	47,741	46,797	801 / 655	42 / 12	962 / 814
AluSq	313	313	19,749	21,263	20,744	354 / 358	39 / 3	496 / 473
FLAM_C	144	320	19,534	21,005	17,250	457 / 372	27 / 10	519 / 363
AluSx4	311	311	9,939	10,846	10,416	166 / 151	29 / 2	201 / 182
AluSc	309	309	30,459	32,870	31,937	433 / 402	25 / 4	535 / 476
AluSg4	312	312	6,365	6,896	6,575	91 / 67	25 / 2	135 / 111
FLAM_A	142	302	12,326	15,783	8,967	186 / 164	18 / 7	201 / 150
AluSg7	312	312	7,189	7,828	7,310	143 / 101	17 / 3	128 / 135
AluSc8	312	312	18,673	21,351	20,351	296 / 297	12 / 7	422 / 333
FRAM	175	320	6,313	7,771	4,755	153 / 122	8 / 5	170 / 116
AluSq10	312	312	2,180	2,226	1,319	71 / 52	12 / 0	24 / 20
AluSc5	312	312	6,110	6,450	5,907	85 / 77	9 / 1	114 / 92
AluSq4	311	311	1,686	1,802	1,688	22 / 24	5 / 1	36 / 35
FAM	185	185	4,036	4,652	3,099	59 / 45	3 / 2	52 / 39
AluYc	299	299	3,766	6,674	6,527	67 / 40	5 / 0	99 / 121
AluYk4	312	312	1,629	1,787	1,766	18 / 22	2 / 0	36 / 26
AluYf5	312	312	165	180	177	0 / 3	1 / 0	4 / 3
AluYb8	318	318	2,516	2,611	2,590	39 / 29	1 / 0	35 / 31
AluYf4	312	312	1,050	1,331	1,303	20 / 12	1 / 0	16 / 15
AluYh9	311	311	129	164	156	0 / 4	0 / 0	3 / 2
AluYd8	299	299	158	199	198	3 / 2	0 / 0	2 / 1
AluYa8	310	310	55	316	307	0 / 1	0 / 0	20 / 8
AluYg6	311	311	543	604	596	10 / 8	0 / 0	10 / 7
AluYc3	300	300	539	539	476	7 / 11	0 / 0	11 / 10
AluYk11	312	312	1,001	1,001	114	14 / 15	0 / 0	2 / 2
AluYa5	310	310	3,459	3,820	3,793	40 / 36	0 / 0	46 / 42

Repeat Map

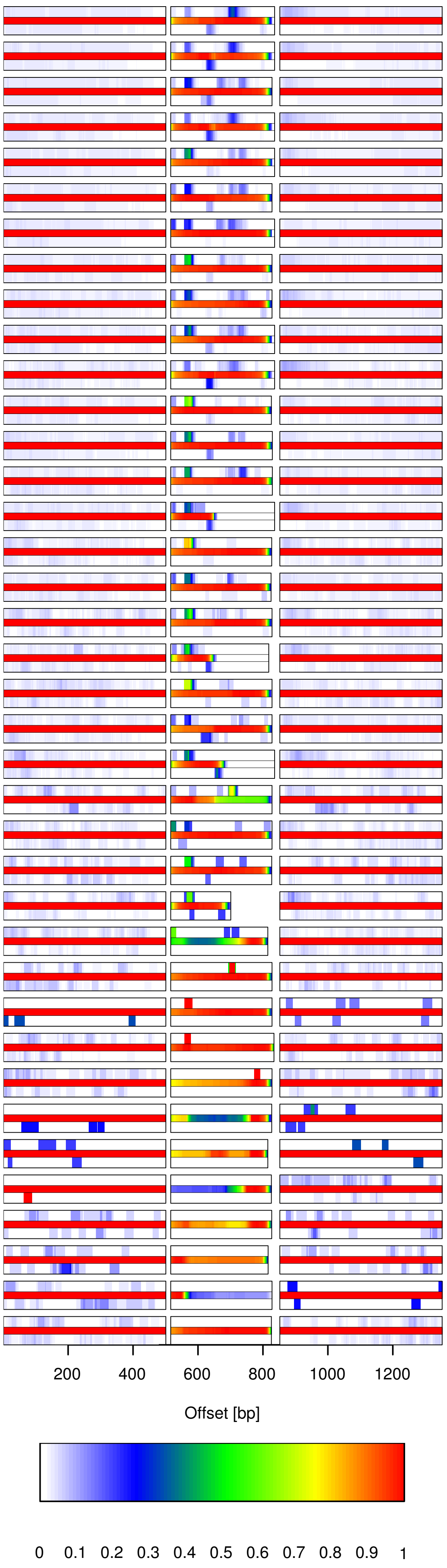

Alu (2/2)

Name	Avg.len.	Max.len.	F5 cov.	Max.cov.	F3 cov.	F5 (+/-)	Rep. (+/-)	F3 (+/-)
AluYb9	318	318	289	319	306	8 / 3	0 / 0	5 / 3
AluYc5	299	299	12	43	43	0 / 0	0 / 0	2 / 1
AluYk12	312	312	126	165	126	1 / 0	0 / 0	3 / 0

(Column groups: Repeat Statistics — Name, Avg.len., Max.len., F5 cov., Max.cov., F3 cov.; Quadruplex Count — F5 (+/-), Rep. (+/-), F3 (+/-))

Repeat Map

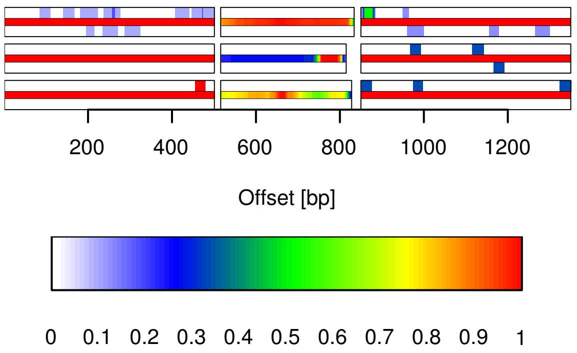

ERVL-MaLR (1/3)

	Repeat Statistics					Quadruplex Count		
Name	Avg.len.	Max.len.	F5 cov.	Max.cov.	F3 cov.	F5 (+/-)	Rep. (+/-)	F3 (+/-)
MSTB	426	595	6,753	6,759	6,579	79 / 81	1,037 / 75	73 / 91
MSTA	428	505	15,726	16,038	15,343	186 / 98	705 / 129	134 / 150
MSTB1	433	505	3,520	3,578	3,513	25 / 33	630 / 45	37 / 39
MLT1A0	368	680	15,758	16,241	15,778	221 / 209	188 / 168	200 / 263
MLT1D	507	680	13,816	13,931	13,620	305 / 307	152 / 131	292 / 287
MLT1K	594	680	4,821	9,059	8,925	142 / 119	95 / 174	191 / 212
THE1D	382	610	10,802	11,065	11,056	128 / 98	194 / 40	106 / 105
THE1B	364	1,651	20,371	20,562	20,409	187 / 199	193 / 25	155 / 190
MLT1A	378	672	6,600	6,676	6,511	132 / 111	73 / 139	89 / 101
MSTB2	456	569	1,209	1,218	1,222	7 / 8	199 / 4	13 / 11
THE1A-int	1,580	1,735	1,123	1,114	1,126	3 / 1	34 / 164	5 / 3
THE1B-int	1,578	1,735	3,218	3,205	3,247	6 / 7	126 / 59	5 / 1
MSTD	398	654	5,766	5,967	5,886	48 / 50	87 / 80	50 / 60
MLT1B	393	680	12,395	12,844	12,528	283 / 302	35 / 126	268 / 303
MLT1C	468	1,387	12,767	14,005	13,551	386 / 370	79 / 80	395 / 448
MLT1J	514	680	6,697	9,020	6,668	102 / 130	35 / 113	96 / 116
MLT1H	544	680	4,979	6,573	5,424	73 / 70	5 / 128	53 / 57
MSTA-int	1,628	1,735	1,901	2,041	2,100	12 / 7	38 / 94	160 / 5
MLT1L	607	680	2,719	5,619	5,490	47 / 36	62 / 56	80 / 68
THE1C	376	1,580	8,843	8,831	8,637	79 / 82	91 / 26	74 / 85
MLT1O	546	672	1,011	2,922	1,940	38 / 14	34 / 54	27 / 37
MLT1J2	450	672	2,906	4,299	3,784	46 / 39	22 / 62	47 / 61
MLT1G1	576	680	1,875	2,305	2,289	21 / 25	9 / 67	32 / 15
THE1C-int	1,582	1,735	1,332	1,319	1,349	0 / 0	45 / 30	0 / 1
THE1A	355	465	3,803	3,833	3,813	24 / 29	68 / 5	24 / 25
MLT1H1	559	680	2,114	2,296	2,123	34 / 28	7 / 64	26 / 26
THE1D-int	1,584	1,735	1,294	1,316	1,343	4 / 3	38 / 32	3 / 4
MLT1A1	408	672	5,098	5,279	5,190	64 / 64	30 / 39	64 / 53
MLT1J1	447	672	2,255	3,259	2,200	29 / 33	10 / 58	18 / 28
MLT1E2	624	680	2,230	2,593	2,444	24 / 23	26 / 41	28 / 24
MLT1F2	563	680	3,884	4,012	3,831	34 / 44	8 / 58	29 / 54
MSTA1	464	465	344	410	415	2 / 4	60 / 5	1 / 6
MLT1H2	509	672	1,634	2,861	2,331	23 / 21	6 / 55	18 / 41
MLT1I	417	1,387	3,744	7,118	6,933	54 / 59	16 / 43	70 / 104
MSTC	427	562	2,272	2,261	2,152	23 / 19	46 / 8	23 / 37
MLT1E1A	666	1,489	1,701	2,058	2,084	24 / 23	14 / 34	17 / 21
MLT1G	585	1,409	1,626	1,774	1,571	31 / 22	7 / 39	26 / 36
MLT1A1-int	1,703	1,735	220	213	226	4 / 0	39 / 3	0 / 0

Repeat Map

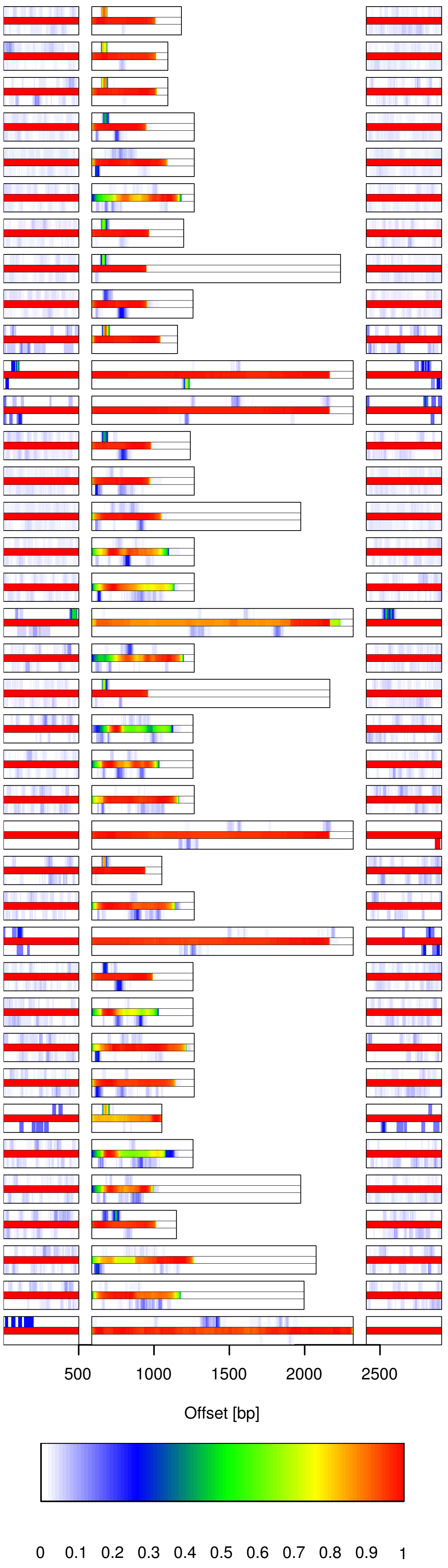

ERVL-MaLR (2/3)

	Repeat Statistics					Quadruplex Count		
Name	Avg.len.	Max.len.	F5 cov.	Max.cov.	F3 cov.	F5 (+/-)	Rep. (+/-)	F3 (+/-)
MLT1F	551	680	2,574	2,824	2,760	49 / 34	8 / 29	24 / 32
MSTD-int	1,725	1,735	355	360	375	0 / 1	13 / 22	1 / 3
MSTB-int	1,648	1,735	543	531	535	1 / 1	12 / 21	1 / 0
MLT1N2	562	672	1,498	3,699	3,762	28 / 19	18 / 11	52 / 49
MLT1F1	568	680	1,808	1,923	1,871	23 / 29	2 / 27	21 / 26
MLT1E1	647	680	554	610	615	3 / 10	2 / 23	6 / 8
MLT1M	668	672	673	1,363	443	11 / 8	12 / 12	6 / 4
MLT1A0-int	1,670	1,735	409	388	388	1 / 1	18 / 6	1 / 0
MLT1E3	629	680	1,004	1,100	1,136	14 / 13	7 / 15	18 / 10
MLT1G3	587	680	1,452	1,648	1,498	22 / 18	3 / 17	28 / 26
MLT1E	649	680	443	519	507	7 / 2	6 / 11	5 / 5
MST-int	1,647	1,735	72	146	66	0 / 0	3 / 13	2 / 1
MLT1J-int	1,388	1,489	335	435	457	2 / 1	10 / 4	2 / 1
MLT1I-int	1,390	1,489	279	274	266	1 / 3	9 / 2	3 / 1
MLT1H-int	1,411	1,735	266	254	234	3 / 1	8 / 3	0 / 1
MLT1J2-int	1,388	1,489	204	200	202	4 / 0	6 / 3	2 / 1
MSTC-int	1,712	1,735	92	91	92	0 / 0	1 / 7	0 / 0
MLT1J1-int	1,387	1,489	170	169	157	2 / 1	7 / 1	0 / 1
MLT1C-int	1,401	1,735	164	184	194	1 / 1	6 / 2	0 / 1
MLT1G3-int	1,454	1,489	72	83	88	0 / 0	6 / 0	0 / 0
THE1-int	1,589	1,735	53	332	65	0 / 0	6 / 0	0 / 1
MSTB1-int	1,648	1,735	104	108	109	0 / 0	3 / 1	0 / 0
MLT1A-int	1,581	1,735	132	135	189	0 / 2	3 / 1	0 / 2
MLT1B-int	1,476	1,735	170	166	193	0 / 0	3 / 1	0 / 8
MLT1G1-int	1,454	1,735	112	130	139	0 / 0	3 / 1	0 / 2
MLT1H1-int	1,429	1,489	157	149	161	1 / 1	4 / 0	2 / 1
MLT1H2-int	1,417	1,735	132	124	130	6 / 0	1 / 2	0 / 0
MLT1F1-int	1,476	1,651	61	64	66	0 / 0	3 / 0	0 / 0
MLT1E2-int	1,450	1,735	48	46	48	0 / 0	3 / 0	0 / 0
MLT1F-int	1,482	1,735	139	170	156	1 / 0	3 / 0	0 / 1
MLT1-int	1,398	1,735	55	75	26	0 / 2	1 / 1	0 / 0
MLT1F2-int	1,477	1,489	107	114	123	0 / 0	1 / 1	0 / 0
MLT1G-int	1,441	1,489	66	67	67	1 / 0	2 / 0	1 / 0
MSTB2-int	1,673	1,735	46	46	46	0 / 0	0 / 1	0 / 0
MLT1D-int	1,413	1,735	243	231	249	0 / 1	1 / 0	0 / 0
MLT-int	1,724	1,735	82	106	85	0 / 2	1 / 0	0 / 1
MLT1E3-int	1,463	1,489	28	29	25	0 / 0	0 / 0	0 / 1
MLT1E1A-int	1,479	1,489	40	37	36	0 / 0	0 / 0	0 / 0

Repeat Map

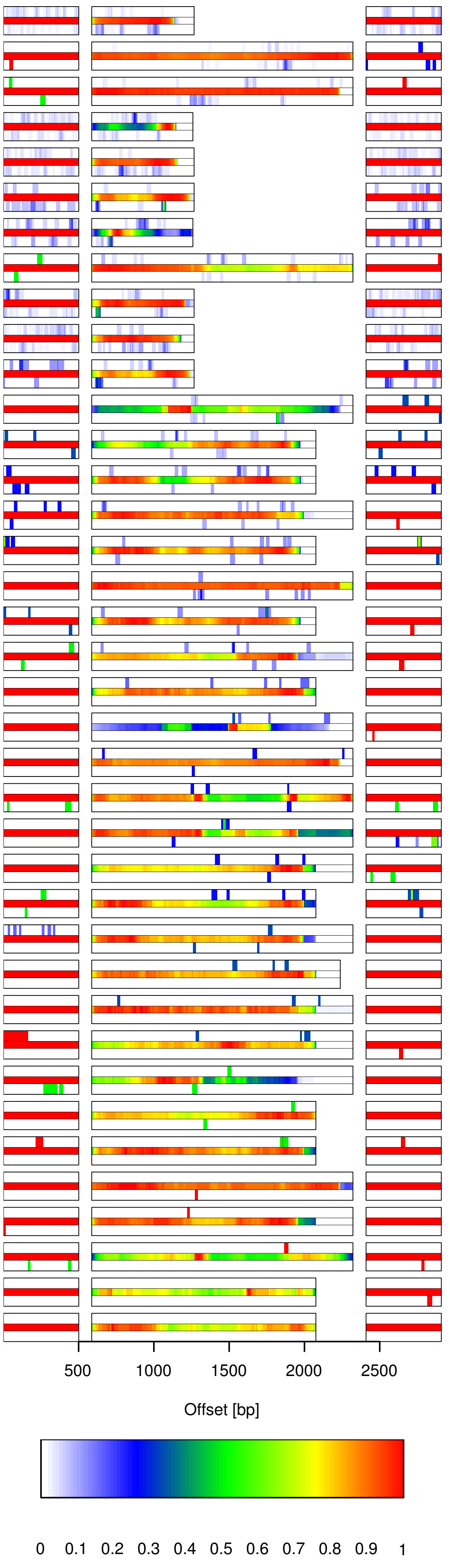

ERVL-MaLR (3/3)

Name	Avg.len.	Max.len.	F5 cov.	Max.cov.	F3 cov.	F5 (+/-)	Rep. (+/-)	F3 (+/-)
MLT1M-int	1,489	1,489	0	1	0	0 / 0	0 / 0	0 / 0
MLT1L-int	1,388	1,409	15	35	35	0 / 0	0 / 0	0 / 0
MLT1E1-int	1,466	1,489	13	17	21	0 / 0	0 / 0	0 / 0
MLT1E-int	1,454	1,489	7	6	8	0 / 0	0 / 0	0 / 0
MLT1O-int	1,388	1,409	3	4	3	0 / 0	0 / 0	0 / 0
MLT1N2-int	1,389	1,489	10	42	42	0 / 0	0 / 0	0 / 0
MSTA1-int	1,654	1,735	10	14	13	0 / 0	0 / 0	0 / 0
MLT1K-int	1,389	1,489	21	83	85	0 / 0	0 / 0	1 / 0

(Repeat Statistics: Name – F3 cov.; Quadruplex Count: F5 (+/-) – F3 (+/-))

Repeat Map

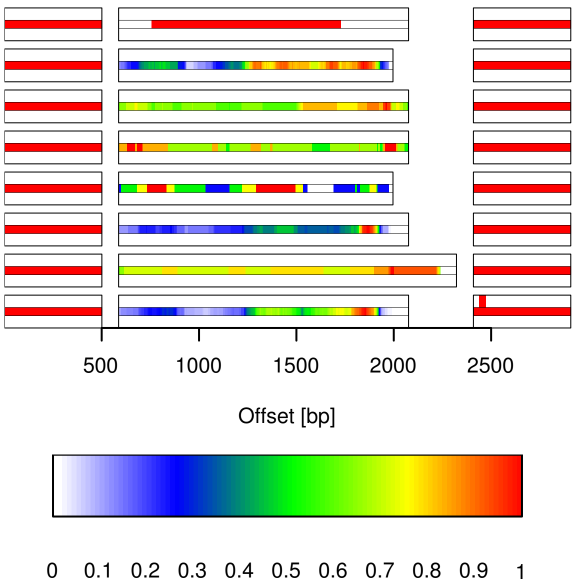

ERVL (1/4)

Name	Avg.len.	Max.len.	F5 cov.	Max.cov.	F3 cov.	F5 (+/-)	Rep. (+/-)	F3 (+/-)
	**Repeat Statistics**					**Quadruplex Count**		
LTR33	515	795	5,153	5,701	4,792	79 / 84	105 / 394	79 / 112
LTR16C	491	651	2,275	4,740	4,564	36 / 34	26 / 313	108 / 100
HERVL-int	5,646	5,757	717	823	921	1 / 0	23 / 237	4 / 4
MER21C	935	938	2,157	3,007	2,343	32 / 27	88 / 150	35 / 56
MER74A	561	901	1,040	1,132	1,039	19 / 29	3 / 196	20 / 29
LTR16A	450	651	2,393	3,166	2,846	30 / 30	12 / 177	51 / 41
LTR16A1	457	651	1,479	1,854	1,796	31 / 18	9 / 160	34 / 37
MER21B	874	938	1,444	1,597	1,364	19 / 14	73 / 78	11 / 31
LTR16A2	495	651	798	1,306	1,240	10 / 19	9 / 118	28 / 28
LTR52	421	421	731	773	653	11 / 11	1 / 120	5 / 3
RV3-16A3_I-int	5,246	5,757	672	1,091	1,362	8 / 12	83 / 35	6 / 17
LTR16	441	568	1,880	2,274	2,075	21 / 27	10 / 95	20 / 30
ERVL-E-int	5,649	6,189	880	1,049	1,132	9 / 9	56 / 46	7 / 10
MER21A	928	938	875	930	822	10 / 20	53 / 46	15 / 7
LTR33A	527	795	854	1,277	1,248	14 / 11	37 / 59	20 / 19
LTR16B2	462	489	973	1,113	1,021	14 / 8	5 / 80	12 / 13
LTR16E1	538	651	869	1,707	1,676	12 / 15	10 / 72	29 / 25
MER74B	621	901	384	624	359	7 / 8	2 / 75	5 / 8
LTR33A_	532	795	899	1,055	895	24 / 14	29 / 42	18 / 10
MER76	691	691	447	452	437	9 / 8	10 / 58	4 / 6
LTR50	789	795	1,296	1,270	904	12 / 16	16 / 42	15 / 14
MER54A	890	901	375	382	302	6 / 4	0 / 57	2 / 4
ERVL-B4-int	5,691	5,757	585	584	718	4 / 4	25 / 30	2 / 6
LTR16E2	568	675	839	1,521	1,442	14 / 14	5 / 49	27 / 28
LTR79	527	527	1,145	2,329	2,019	22 / 7	21 / 32	26 / 21
MLT2B2	525	717	1,446	1,444	1,348	12 / 17	5 / 45	15 / 14
MLT2B3	567	729	2,139	2,202	2,242	13 / 19	9 / 38	19 / 33
MER73	661	901	187	188	166	3 / 3	0 / 40	1 / 2
HERVL18-int	6,047	6,047	102	118	80	3 / 0	25 / 13	0 / 0
LTR41B	756	795	385	400	384	5 / 4	13 / 25	4 / 4
LTR47A	452	464	479	510	463	5 / 8	22 / 14	6 / 3
LTR16B1	479	480	770	929	870	8 / 7	4 / 31	9 / 14
HERVL40-int	5,416	6,189	145	158	193	0 / 0	13 / 20	1 / 1
HERV16-int	5,090	5,757	607	423	38	2 / 8	23 / 10	0 / 0
LTR32	457	457	817	934	808	15 / 9	13 / 15	20 / 14
LTR16D	650	675	177	675	671	2 / 0	3 / 25	8 / 10
MLT2B1	531	601	2,741	2,830	2,637	18 / 20	9 / 19	40 / 31
MER68	565	938	973	1,063	1,029	8 / 10	14 / 13	8 / 18

Repeat Map

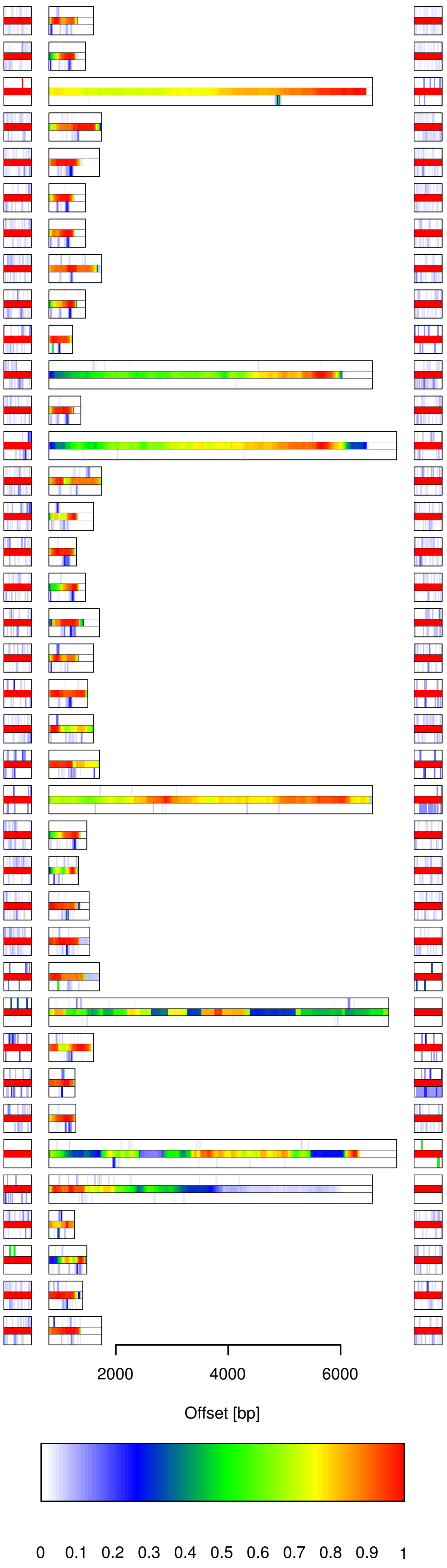

ERVL (2/4)

Name	Avg.len.	Max.len.	F5 cov.	Max.cov.	F3 cov.	F5 (+/-)	Rep. (+/-)	F3 (+/-)
	**Repeat Statistics**					**Quadruplex Count**		
TR101_Mam	829	829	379	545	281	10 / 8	22 / 3	7 / 2
MLT2F	655	663	1,071	1,146	1,019	11 / 6	11 / 13	2 / 8
TR105_Mam	600	600	315	384	219	4 / 5	9 / 15	2 / 3
MER77	624	938	798	857	790	13 / 10	8 / 16	7 / 16
MLT2D	446	717	2,446	2,671	2,317	36 / 41	2 / 22	32 / 45
MER21-int	4,211	6,047	124	180	210	0 / 0	21 / 2	2 / 0
LTR16B	463	480	445	517	486	6 / 2	1 / 21	7 / 7
MER77B	737	938	733	805	589	10 / 7	12 / 10	6 / 7
LTR67B	618	620	1,622	1,941	1,533	21 / 25	9 / 13	21 / 28
MLT2B4	554	729	2,505	2,573	2,215	43 / 35	10 / 11	25 / 26
LTR18C	356	614	65	71	67	1 / 0	0 / 19	0 / 0
LTR33C	626	663	516	533	461	13 / 1	0 / 18	7 / 4
MER54B	806	901	227	264	197	1 / 3	6 / 11	3 / 4
LTR40b	466	584	597	831	840	6 / 5	13 / 3	8 / 6
LTR40a	518	608	1,263	1,377	1,289	14 / 16	14 / 2	7 / 17
LTR41	766	795	484	573	595	3 / 2	8 / 8	4 / 4
LTR53B	533	533	149	155	146	1 / 6	0 / 15	2 / 5
LTR18B	610	614	274	295	276	2 / 4	1 / 14	2 / 3
V3-16A3_LTR	428	480	690	759	626	5 / 11	3 / 12	16 / 9
LTR33B	507	795	702	886	825	12 / 10	4 / 9	12 / 4
HERVL32-int	3,790	6,047	27	38	27	0 / 0	11 / 1	0 / 0
TR102_Mam	851	851	196	255	248	0 / 2	4 / 7	5 / 3
LTR84b	761	780	428	474	455	7 / 9	1 / 10	10 / 6
LTR53	519	533	353	384	376	5 / 4	1 / 10	4 / 5
LTR42	495	495	212	228	208	3 / 9	2 / 9	2 / 12
LTR40c	473	584	420	476	404	7 / 4	7 / 3	8 / 7
LTR75	564	608	189	194	162	7 / 2	1 / 9	3 / 4
LTR53-int	3,512	5,685	66	76	42	0 / 2	2 / 7	0 / 0
MER68B	565	700	298	296	251	6 / 9	6 / 3	6 / 2
LTR82B	832	872	392	526	442	4 / 5	2 / 7	8 / 5
LTR86A1	513	513	245	311	214	1 / 1	3 / 6	5 / 2
LTR16D2	585	675	123	165	162	0 / 0	0 / 9	1 / 1
LTR41C	712	795	404	395	341	2 / 3	7 / 2	4 / 6
LTR16D1	667	675	55	128	120	0 / 1	1 / 7	0 / 1
MLT2C1	425	663	1,576	1,656	1,159	31 / 22	2 / 6	13 / 29
MLT2C2	483	567	721	748	618	13 / 13	1 / 6	9 / 9
MLT2A2	505	717	3,130	3,115	3,118	23 / 24	5 / 2	36 / 28
ERVL-int	5,716	5,757	43	166	244	0 / 0	4 / 3	0 / 0

Repeat Map

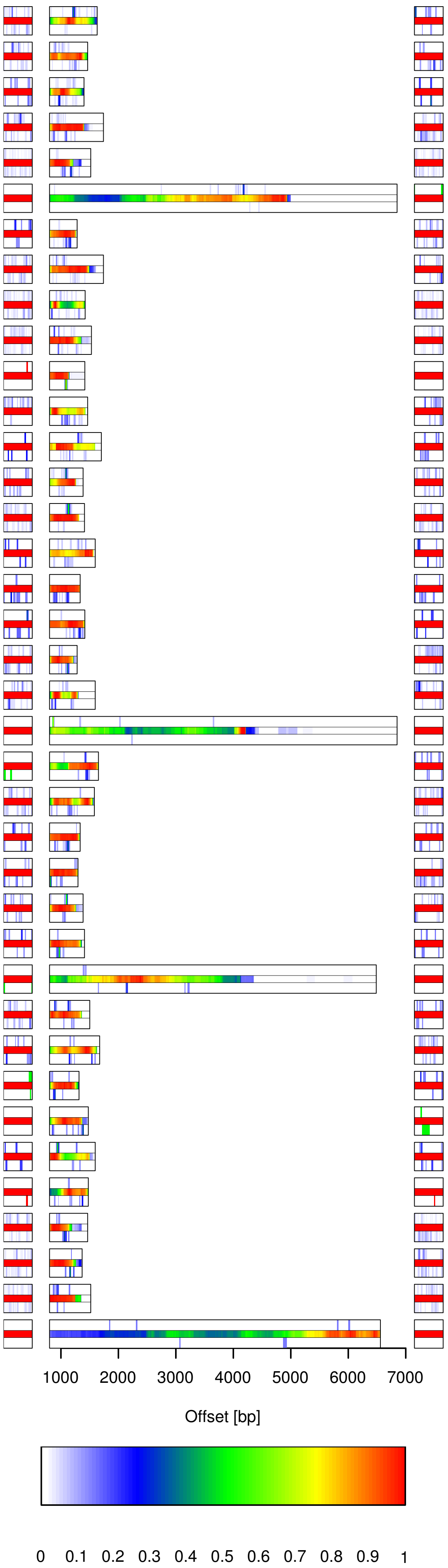

ERVL (3/4)

Name	Avg.len.	Max.len.	F5 cov.	Max.cov.	F3 cov.	F5 (+/-)	Rep. (+/-)	F3 (+/-)
	**Repeat Statistics**					**Quadruplex Count**		
MER68-int	3,018	5,530	69	88	82	0 / 0	4 / 3	0 / 2
LTR18A	366	614	176	176	173	1 / 1	0 / 6	0 / 1
MER74C	469	622	174	177	153	0 / 0	0 / 6	1 / 3
MER70A	475	578	417	459	447	2 / 2	2 / 4	4 / 3
MER88	475	901	80	87	57	1 / 0	0 / 5	0 / 1
LTR40A1	581	584	268	320	133	5 / 2	1 / 4	0 / 0
HERVL74-int	5,489	5,685	23	49	45	0 / 0	1 / 4	0 / 1
MER70C	540	578	185	205	169	1 / 1	5 / 0	2 / 1
MLT2E	622	663	210	212	176	4 / 1	0 / 5	2 / 3
LTR86A2	508	513	161	183	105	3 / 3	3 / 2	0 / 1
LTR83	780	780	240	291	273	5 / 3	4 / 1	2 / 8
ERVL47-int	5,250	6,047	36	33	35	0 / 0	3 / 1	0 / 0
MER70-int	5,023	5,530	41	67	74	0 / 0	2 / 2	0 / 1
LTR57	411	434	194	203	189	3 / 3	3 / 1	2 / 3
MLT2B5	660	717	182	204	192	1 / 3	0 / 4	1 / 2
LTR82A	870	872	436	471	360	6 / 2	2 / 2	6 / 7
MER70B	574	578	305	338	92	4 / 4	0 / 4	1 / 1
MLT2A1	470	601	2,396	2,404	2,285	19 / 23	1 / 3	22 / 17
LTR21C	511	511	18	24	22	1 / 0	0 / 4	0 / 0
MER76-int	6,158	6,189	31	27	21	0 / 0	1 / 2	0 / 0
LTR80A	583	584	219	253	245	1 / 0	3 / 0	1 / 2
LTR84a	757	761	214	258	249	3 / 1	0 / 3	8 / 1
HERVL66-int	4,329	4,329	25	27	24	0 / 0	3 / 0	0 / 0
LTR57-int	5,342	5,343	88	84	20	0 / 0	2 / 1	0 / 0
LTR86B2	487	493	130	169	115	3 / 0	2 / 0	2 / 0
LTR86B1	493	493	131	178	145	1 / 2	2 / 0	1 / 2
LTR62	709	709	199	197	194	7 / 9	0 / 2	16 / 9
LTR52-int	3,650	5,685	64	54	31	0 / 0	0 / 1	0 / 0
LTR69	588	588	104	104	101	0 / 1	0 / 1	1 / 0
LTR80B	584	584	214	339	307	1 / 0	0 / 1	2 / 1
LTR47A2	432	452	164	178	157	3 / 2	1 / 0	2 / 4
LTR75B	606	608	98	153	142	2 / 3	0 / 1	2 / 1
LTR86C	621	621	135	153	138	0 / 2	1 / 0	2 / 2
LTR47B4	434	434	73	86	83	1 / 3	0 / 0	0 / 1
MER68C	421	548	101	110	101	3 / 0	0 / 0	0 / 0
LTR91	199	199	94	341	217	2 / 3	0 / 0	3 / 0
LTR47B2	435	464	129	135	123	1 / 0	0 / 0	2 / 3
LTR66	610	610	147	262	141	4 / 1	0 / 0	3 / 2

Repeat Map

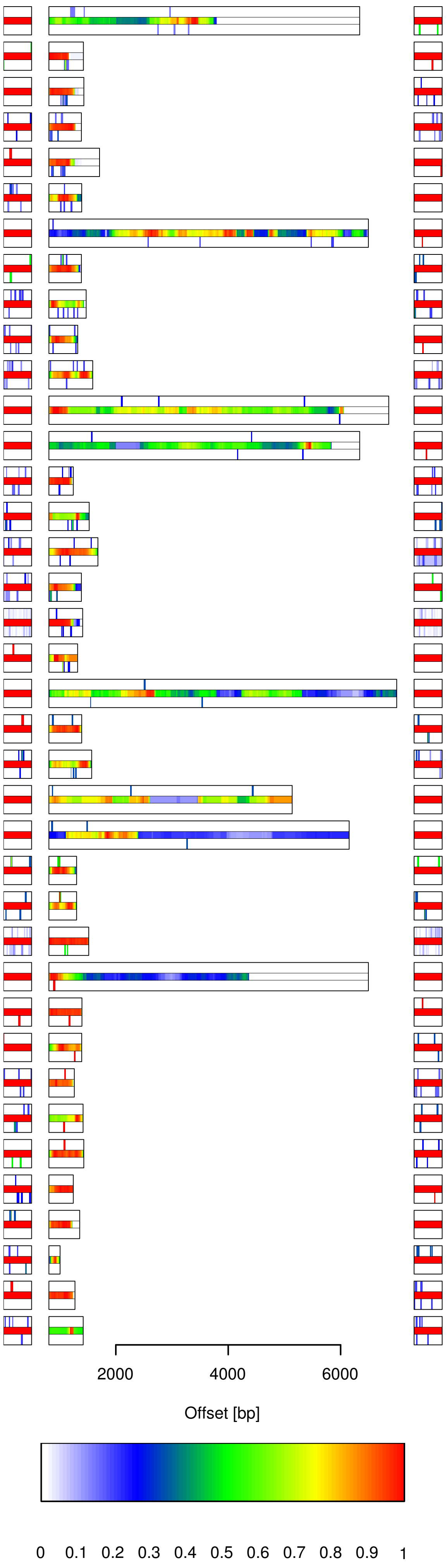

ERVL (4/4)

Repeat Statistics						Quadruplex Count		
Name	Avg.len.	Max.len.	F5 cov.	Max.cov.	F3 cov.	F5 (+/-)	Rep. (+/-)	F3 (+/-)
TR108b_Mam	501	544	33	35	34	0 / 0	0 / 0	1 / 0
TR108c_Mam	519	519	25	31	30	0 / 0	0 / 0	0 / 0
LTR47B	443	452	31	31	30	2 / 1	0 / 0	1 / 1
TR108d_Mam	544	548	42	48	44	0 / 0	0 / 0	0 / 0
TR108e_Mam	548	548	84	104	89	1 / 2	0 / 0	1 / 0
LTR47B3	463	464	68	82	72	0 / 0	0 / 0	2 / 0
TR108a_Mam	511	519	53	57	51	0 / 0	0 / 0	0 / 0

Repeat Map

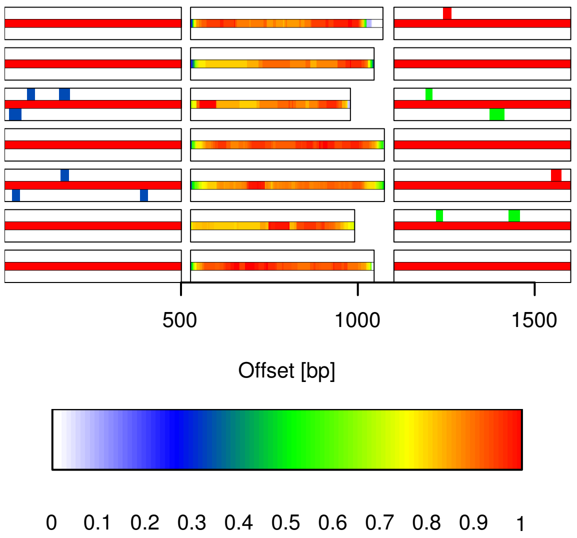

ERV1 (1/8)

	Repeat Statistics					Quadruplex Count		
Name	Avg.len.	Max.len.	F5 cov.	Max.cov.	F3 cov.	F5 (+/-)	Rep. (+/-)	F3 (+/-)
LTR12C	1,577	1,579	2,406	2,416	2,429	45 / 32	1,555 / 393	47 / 24
HERVH-int	7,700	8,919	1,111	1,121	1,153	2 / 0	3 / 799	6 / 2
MER52A	1,770	2,123	958	1,033	1,038	9 / 5	221 / 187	6 / 7
HERV9-int	8,346	8,919	364	326	347	0 / 0	236 / 113	0 / 1
LTR1A2	837	1,322	653	646	615	19 / 6	243 / 65	6 / 6
LTR12D	1,261	1,579	245	386	363	8 / 0	272 / 9	4 / 5
MER52D	2,077	2,123	267	321	260	7 / 4	102 / 127	2 / 3
LTR8B	816	820	1,047	1,041	1,041	14 / 14	4 / 210	16 / 16
LTR78	1,304	1,304	926	1,516	1,568	11 / 13	104 / 87	23 / 30
LTR8	695	820	2,245	2,224	2,066	22 / 22	3 / 186	25 / 35
LTR1A1	799	974	304	308	292	6 / 1	134 / 53	1 / 3
LTR1E	812	975	253	259	256	5 / 0	24 / 127	1 / 1
LTR7	451	779	2,230	2,268	2,262	18 / 5	0 / 146	16 / 20
LTR1D1	901	1,020	261	257	246	1 / 4	32 / 110	3 / 7
LTR12	835	1,579	701	703	456	9 / 13	126 / 7	6 / 9
LTR12_	698	1,579	455	470	403	2 / 4	122 / 7	6 / 4
LTR1	786	975	196	197	164	5 / 5	83 / 44	1 / 4
LTR8A	730	820	934	925	911	14 / 13	3 / 122	12 / 15
MER61A	341	427	729	733	719	6 / 5	6 / 102	11 / 5
LTR1D	973	1,278	252	252	249	0 / 1	12 / 94	3 / 3
LTR78B	1,296	1,304	688	1,024	1,109	10 / 15	58 / 45	19 / 16
MER52C	1,303	2,123	237	285	282	5 / 2	18 / 71	1 / 0
LTR12E	1,345	1,579	84	89	57	0 / 1	55 / 32	2 / 1
ERVFH21-int	6,605	8,436	36	32	21	0 / 1	0 / 78	0 / 1
LTR30	722	722	124	123	120	0 / 1	19 / 57	1 / 0
MER4E1	781	903	568	630	648	9 / 4	5 / 70	15 / 16
MER39	703	787	1,213	1,827	1,805	11 / 14	3 / 66	34 / 17
LTR26	603	820	388	428	425	5 / 15	2 / 66	14 / 7
MER4C	469	872	451	482	481	7 / 5	0 / 65	7 / 10
LTR46	460	460	186	186	174	10 / 2	0 / 62	15 / 13
MER50	734	782	1,557	1,679	1,570	25 / 19	15 / 47	32 / 21
HERV17-int	8,610	8,626	170	227	236	0 / 1	3 / 57	0 / 0
MER4D1	889	923	791	803	814	22 / 12	13 / 44	6 / 19
LTR48	772	787	492	515	527	5 / 4	1 / 55	4 / 4
LTR1B1	825	2,123	133	138	140	0 / 4	31 / 24	2 / 0
MER61B	430	1,077	356	377	374	3 / 4	22 / 32	2 / 1
LTR48B	651	900	619	617	594	7 / 10	2 / 51	5 / 7
MER4B	639	923	960	957	837	13 / 14	6 / 46	10 / 23

Repeat Map

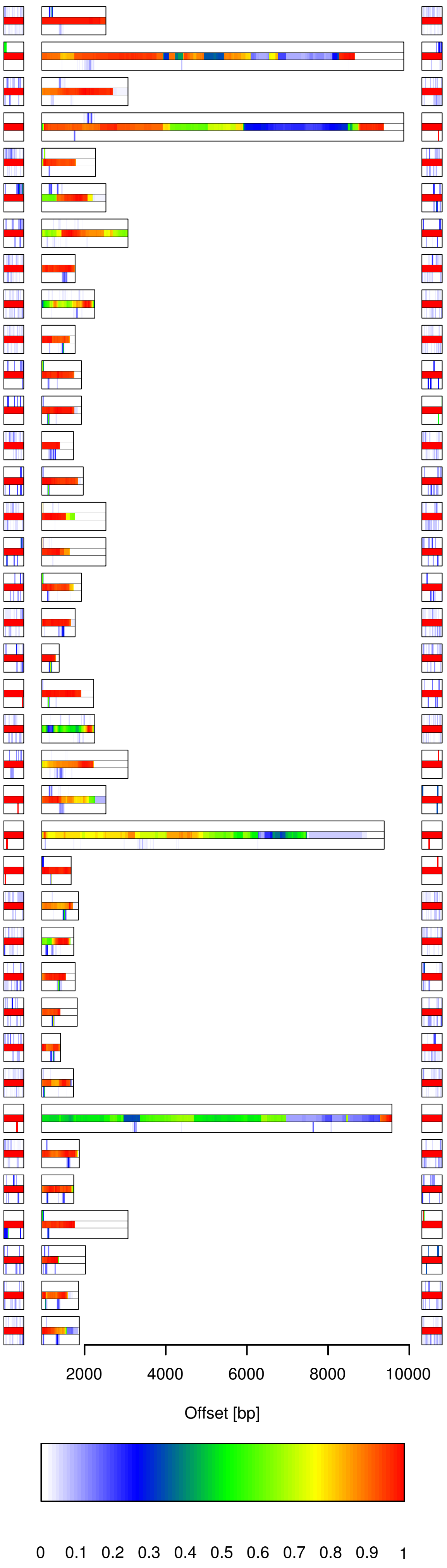

ERV1 (2/8)

Repeat Statistics						Quadruplex Count		
Name	Avg.len.	Max.len.	F5 cov.	Max.cov.	F3 cov.	F5 (+/-)	Rep. (+/-)	F3 (+/-)
LTR2C	497	501	167	224	221	4 / 2	0 / 51	8 / 13
MER41-int	6,163	8,270	349	285	302	4 / 0	40 / 9	1 / 2
LTR9B	645	844	408	531	379	10 / 1	18 / 30	4 / 5
LTR1B	822	837	266	283	288	2 / 5	9 / 38	0 / 1
LTR1F1	731	824	185	185	173	2 / 6	9 / 37	0 / 4
MER67B	649	711	281	286	228	10 / 8	1 / 45	3 / 12
MER61-int	5,384	8,919	237	252	265	1 / 0	9 / 34	0 / 1
MER67A	548	711	264	264	266	7 / 7	0 / 43	3 / 3
LTR10D	514	688	155	158	158	9 / 2	3 / 37	1 / 3
MER52-int	7,033	8,919	141	121	102	0 / 0	5 / 35	0 / 0
LTR1F2	744	975	115	117	111	1 / 1	8 / 31	1 / 1
LTR71B	610	610	143	163	152	10 / 6	0 / 39	3 / 3
MER31A	485	661	886	991	931	24 / 26	0 / 39	23 / 34
LTR2752	1,308	2,123	149	177	183	6 / 8	11 / 27	0 / 2
MER4CL34	758	872	401	398	389	15 / 8	2 / 36	7 / 10
MER31B	473	661	1,042	1,041	895	24 / 27	0 / 36	15 / 22
MER4B-int	6,744	8,270	175	152	167	0 / 0	34 / 2	0 / 1
LTR28C	1,042	2,123	271	274	201	6 / 1	11 / 24	2 / 1
MER34A	575	787	818	836	837	11 / 9	1 / 34	17 / 13
MER4-int	6,652	9,044	482	416	335	1 / 0	24 / 10	0 / 1
ERVIP10F-int	7,606	8,978	108	97	109	0 / 0	10 / 24	0 / 0
MER4E	759	923	460	503	508	9 / 4	1 / 33	9 / 3
MER90a	612	669	469	782	765	6 / 9	0 / 33	9 / 15
LTR12F	528	1,254	445	547	477	4 / 9	15 / 18	15 / 7
HERV3-int	8,352	8,978	80	73	59	0 / 2	2 / 30	0 / 1
LTR46-int	6,976	8,626	17	23	25	0 / 0	0 / 31	0 / 1
MER34C2	568	787	347	323	290	3 / 6	2 / 29	6 / 7
MER49	920	923	681	728	571	10 / 18	1 / 29	19 / 20
LTR21A	481	481	80	85	84	0 / 1	0 / 29	2 / 2
LTR39	783	783	625	637	640	16 / 17	5 / 24	11 / 9
MER90	579	669	386	415	411	9 / 6	0 / 28	7 / 10
MER92B	634	636	426	454	404	6 / 3	2 / 26	2 / 5
LTR27C	766	1,755	159	162	137	1 / 3	11 / 17	4 / 3
MER39B	654	707	709	723	709	11 / 7	3 / 25	10 / 14
MER4A	672	900	527	529	500	10 / 10	4 / 24	10 / 13
HERVH48-int	6,653	6,654	35	41	42	0 / 0	0 / 27	0 / 0
MER41B	633	872	1,589	1,993	1,588	17 / 31	3 / 24	34 / 33
MER4D	898	923	556	765	779	10 / 10	5 / 21	15 / 14

Repeat Map

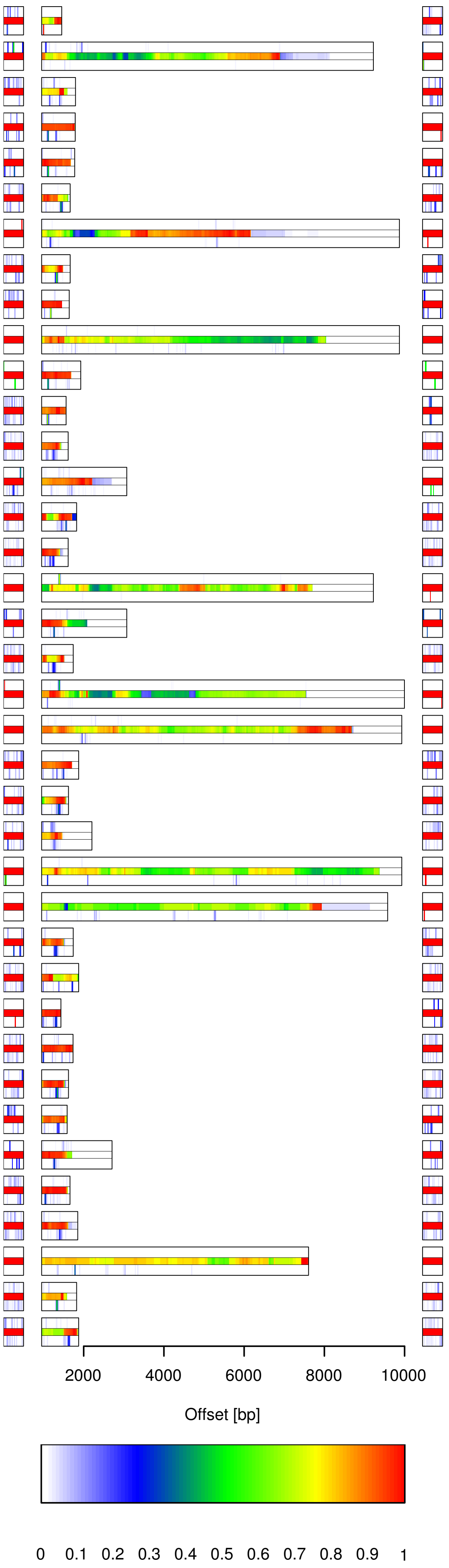

ERV1 (3/8)

	Repeat Statistics					Quadruplex Count		
Name	Avg.len.	Max.len.	F5 cov.	Max.cov.	F3 cov.	F5 (+/-)	Rep. (+/-)	F3 (+/-)
UERS-P2-int	3,332	7,091	89	80	85	0 / 0	7 / 19	0 / 0
LTR1F	774	974	117	117	111	0 / 0	3 / 22	3 / 0
LTR49	601	903	824	858	790	12 / 7	1 / 23	3 / 9
ERVIP10FH-int	5,509	8,978	102	292	81	0 / 0	21 / 3	0 / 0
MER87B	511	556	135	137	129	2 / 2	0 / 23	8 / 0
LTR28B	974	2,123	219	217	206	2 / 1	1 / 22	1 / 1
PRIMA41-int	7,602	8,978	60	68	75	0 / 0	4 / 18	0 / 2
MER51A	630	657	670	667	646	13 / 13	3 / 19	13 / 17
MER89	559	559	766	909	912	15 / 14	1 / 21	29 / 21
LTR23	439	576	362	526	477	1 / 6	3 / 18	9 / 7
HERVI-int	7,717	8,436	51	46	47	0 / 0	2 / 19	0 / 0
MER4A1	476	900	1,679	1,745	1,723	43 / 24	9 / 12	33 / 31
HERVE_a-int	7,821	8,426	105	100	66	0 / 0	6 / 13	0 / 2
LTR25-int	7,199	8,919	90	88	108	0 / 0	7 / 12	0 / 2
LTR1B0	745	899	83	85	83	1 / 0	6 / 13	2 / 0
LTR31	619	722	94	186	179	1 / 5	0 / 19	3 / 3
LTR27B	665	1,263	134	133	97	3 / 1	3 / 16	0 / 1
UERS-P3-int	8,741	8,919	120	135	144	0 / 0	4 / 15	1 / 0
LTR25	842	844	120	176	165	1 / 0	14 / 4	0 / 0
ERVFH19-int	7,575	7,719	26	27	34	1 / 0	0 / 18	0 / 0
MER66-int	6,768	8,919	112	128	157	0 / 1	1 / 17	1 / 0
MER87	541	543	111	118	111	1 / 6	0 / 17	1 / 0
LTR26C	543	622	192	194	185	6 / 4	0 / 17	3 / 4
MER51E	503	657	134	168	129	4 / 6	16 / 1	3 / 9
LTR27E	1,076	1,263	159	163	164	5 / 0	7 / 9	1 / 3
HERVS71-int	8,973	8,978	54	48	47	0 / 0	0 / 16	0 / 1
MER4D0	887	6,598	223	216	218	2 / 3	2 / 14	2 / 3
LTR26E	619	622	70	81	79	1 / 1	1 / 15	0 / 4
LTR36	611	711	299	323	322	2 / 7	1 / 15	6 / 4
MER67C	672	711	862	915	765	14 / 21	5 / 11	16 / 11
RV-Fc1_LTR1	346	346	17	17	17	0 / 0	0 / 15	0 / 2
LTR38B	611	711	146	154	145	7 / 4	0 / 15	4 / 0
HERV4_I-int	6,765	8,919	46	61	85	0 / 2	4 / 11	0 / 1
LTR29	627	872	289	407	377	3 / 6	0 / 15	4 / 5
LTR9D	646	728	495	539	511	11 / 3	2 / 13	7 / 5
LTR19A	487	580	340	361	363	1 / 3	0 / 15	9 / 7
MER34-int	8,034	8,207	68	79	108	0 / 0	1 / 13	0 / 0
MER110	489	614	224	531	494	1 / 3	0 / 14	5 / 1

Repeat Map

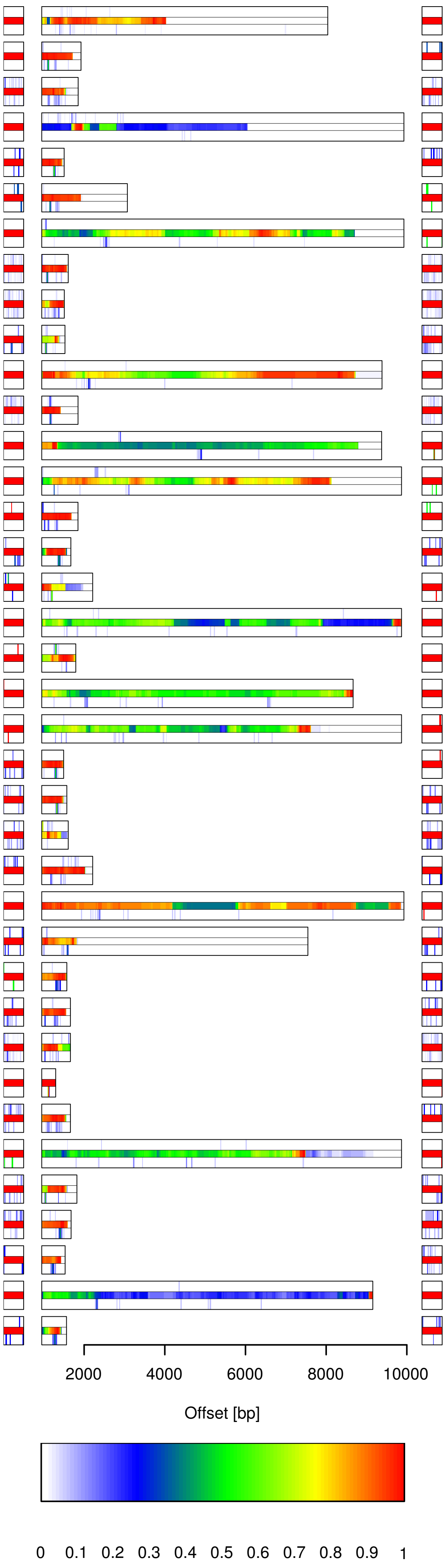

Name	Avg.len.	Max.len.	F5 cov.	Max.cov.	F3 cov.	F5 (+/-)	Rep. (+/-)	F3 (+/-)
MER50-int	7,100	8,919	143	125	96	0 / 0	7 / 7	1 / 0
LTR7Y	471	472	196	228	230	6 / 0	0 / 14	2 / 0
MER57A-int	6,176	9,044	237	188	259	0 / 1	3 / 11	0 / 0
MER51C	639	640	114	123	124	3 / 5	6 / 7	4 / 3
LTR7C	471	472	277	291	280	7 / 2	0 / 13	6 / 4
LTR7B	464	472	643	717	704	9 / 7	0 / 13	9 / 11
MER72	724	923	476	484	492	5 / 5	3 / 9	2 / 6
MER34A1	586	787	438	441	438	8 / 6	0 / 12	10 / 6
LTR72	519	519	109	108	102	1 / 6	1 / 10	4 / 2
PRIMA4-int	8,158	9,044	189	169	35	4 / 4	0 / 11	0 / 0
HERVE-int	7,786	8,426	53	148	44	0 / 0	7 / 4	0 / 2
MER34	546	787	828	829	676	10 / 13	3 / 8	7 / 6
MER66B	487	555	866	1,080	864	8 / 23	3 / 8	13 / 17
MER61C	442	1,263	230	267	253	0 / 0	7 / 3	2 / 5
UERS-P3b-int	7,642	8,919	73	74	83	0 / 0	5 / 5	0 / 1
LTR21B	440	481	23	36	35	0 / 0	0 / 10	1 / 0
MER34C_	584	872	338	428	293	5 / 5	0 / 10	1 / 7
MER50B	698	734	467	493	457	4 / 1	5 / 4	0 / 7
ERVP71A-int	7,578	7,590	53	50	55	0 / 1	6 / 3	0 / 0
MER67D	523	711	636	672	586	3 / 2	1 / 8	11 / 3
LTR19-int	5,677	7,816	35	37	45	0 / 0	1 / 8	0 / 1
MER65C	462	570	454	504	479	6 / 15	6 / 3	8 / 5
MER57-int	7,312	8,270	180	227	299	2 / 0	6 / 3	3 / 2
LTR26D	604	622	124	123	119	6 / 3	0 / 9	1 / 0
Harlequin-int	6,924	7,847	157	151	133	0 / 0	3 / 6	0 / 1
LTR49-int	6,437	9,044	177	228	228	2 / 2	2 / 7	1 / 1
MER31-int	5,169	8,207	183	137	161	2 / 3	0 / 9	1 / 1
LTR38A1	544	608	62	122	122	0 / 4	0 / 8	0 / 0
LTR10F	512	644	349	391	358	3 / 4	0 / 8	6 / 3
MER51B	389	664	534	598	558	13 / 8	2 / 6	11 / 14
LTR38	561	711	147	179	171	5 / 6	0 / 8	3 / 2
LTR1C1	648	648	61	62	61	0 / 0	3 / 4	0 / 1
LTR75_1	546	572	80	87	81	3 / 2	0 / 7	3 / 3
LTR19B	581	779	207	221	221	10 / 1	7 / 0	3 / 2
MER41A	556	781	1,744	1,937	1,685	42 / 38	4 / 3	24 / 32
LOR1a	496	539	565	661	660	8 / 8	0 / 7	7 / 6
MER83	448	448	333	345	324	10 / 4	5 / 2	2 / 5
UERS-P1-int	6,281	8,436	116	127	139	0 / 0	3 / 4	0 / 0

(Column groups: Repeat Statistics — Name, Avg.len., Max.len., F5 cov., Max.cov., F3 cov.; Quadruplex Count — F5 (+/-), Rep. (+/-), F3 (+/-).)

Repeat Map

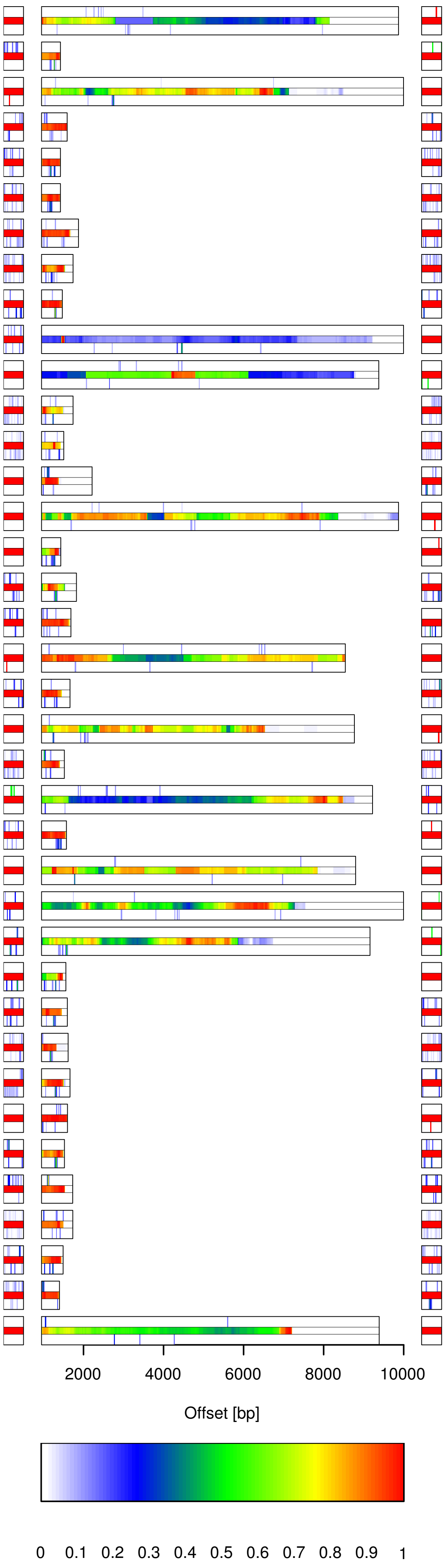

ERV1 (5/8)

Repeat Statistics						Quadruplex Count		
Name	Avg.len.	Max.len.	F5 cov.	Max.cov.	F3 cov.	F5 (+/-)	Rep. (+/-)	F3 (+/-)
LTR70	1,186	1,286	81	80	54	0 / 0	1 / 6	0 / 0
LTR27D	679	1,755	172	181	168	0 / 1	2 / 5	0 / 0
LTR12B	680	1,579	133	141	23	1 / 0	4 / 3	0 / 0
MER51-int	7,615	8,120	104	87	70	0 / 0	6 / 1	1 / 0
PABL_B-int	6,937	9,044	27	40	40	0 / 0	3 / 4	0 / 0
RIMA4_LTR	594	664	228	240	241	2 / 0	1 / 6	3 / 2
HERV15-int	8,659	8,801	21	24	23	0 / 0	1 / 5	0 / 0
LTR35B	603	679	53	142	94	4 / 2	0 / 6	1 / 1
LTR9C	667	779	103	106	97	1 / 0	1 / 5	2 / 1
MER83B	379	448	92	114	104	1 / 0	6 / 0	1 / 2
HERV35I-int	6,761	9,044	53	81	77	0 / 0	1 / 5	0 / 0
MER41G	885	885	31	45	46	1 / 1	3 / 3	1 / 6
LTR60B	759	782	133	148	148	3 / 0	5 / 1	1 / 2
LTR56	448	1,286	618	628	572	20 / 17	0 / 6	7 / 6
MER34B	566	707	398	431	428	4 / 4	0 / 6	1 / 7
RV24_Prim-int	7,494	8,270	41	48	39	0 / 0	1 / 5	1 / 0
MER61D	508	1,020	150	229	218	2 / 2	5 / 1	1 / 6
LTR45C	539	622	255	257	254	8 / 4	0 / 6	5 / 3
LOR1-int	7,796	9,044	194	154	154	4 / 0	1 / 5	0 / 1
MER92-int	5,121	8,270	83	116	73	1 / 1	2 / 4	1 / 1
RV-Fc1_LTR3	476	476	5	5	5	0 / 0	0 / 5	0 / 0
LTR10G	527	609	94	102	99	2 / 2	0 / 5	2 / 1
MER83A-int	4,168	9,044	28	28	15	0 / 1	0 / 5	0 / 0
MER61E	624	1,129	237	237	186	0 / 2	5 / 0	1 / 1
LTR28	1,012	1,129	140	140	112	2 / 1	0 / 5	1 / 1
LTR43	607	765	215	237	229	3 / 5	0 / 5	2 / 2
LTR27	644	1,755	93	100	72	1 / 1	0 / 5	1 / 1
LTR17	780	780	672	767	595	4 / 3	2 / 3	6 / 14
LTR37-int	4,144	6,801	168	159	163	3 / 0	0 / 5	3 / 1
LTR15	498	675	200	222	212	5 / 3	0 / 5	2 / 5
MER57B2	403	435	722	878	762	9 / 6	4 / 1	16 / 4
LTR9A1	727	779	343	343	336	7 / 3	3 / 2	4 / 8
MER110A	474	614	304	401	388	2 / 5	0 / 5	4 / 4
MER84-int	5,051	7,756	19	51	31	0 / 1	1 / 4	0 / 1
LTR1C	747	747	53	54	55	0 / 1	1 / 4	0 / 0
MER48	407	407	173	177	167	5 / 3	1 / 4	1 / 4
LTR37A	428	468	740	1,292	1,170	11 / 8	1 / 4	21 / 19
MER66C	555	634	257	442	292	1 / 3	2 / 2	0 / 2

Repeat Map

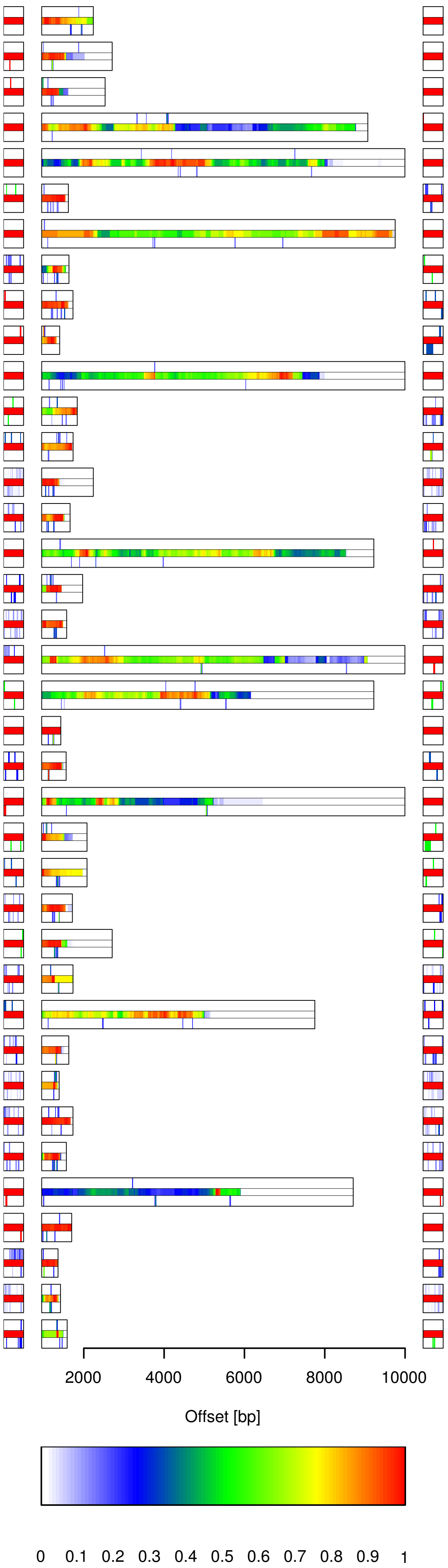

ERV1 (6/8)

Repeat Statistics						Quadruplex Count		
Name	Avg.len.	Max.len.	F5 cov.	Max.cov.	F3 cov.	F5 (+/-)	Rep. (+/-)	F3 (+/-)
IERV-Fc2-int	6,376	7,250	2	2	1	0 / 0	0 / 4	0 / 0
MER92D	330	636	147	173	169	0 / 2	0 / 4	0 / 2
MER61F	675	779	109	115	116	2 / 0	3 / 1	1 / 3
MER83B-int	5,237	8,257	36	43	30	0 / 0	2 / 2	0 / 0
PABL_A-int	5,466	7,816	59	88	74	0 / 0	2 / 2	3 / 0
MER89-int	6,814	8,270	66	105	141	0 / 0	0 / 4	0 / 0
LTR35	604	608	56	66	60	0 / 2	0 / 4	0 / 0
LTR35A	549	608	53	64	61	1 / 1	0 / 4	1 / 0
LOR1b	462	497	785	850	844	7 / 11	0 / 4	15 / 11
LTR77	622	622	83	89	84	0 / 0	2 / 2	0 / 0
LTR10B2	518	633	37	94	85	1 / 1	0 / 4	0 / 0
HERV30-int	8,275	8,626	24	24	24	0 / 0	0 / 3	0 / 0
HERV1_I-int	8,727	8,801	15	16	17	0 / 0	1 / 2	0 / 0
LTR59	599	768	69	73	67	0 / 0	1 / 2	1 / 1
ERV1_LTRa	521	521	23	24	23	0 / 0	0 / 3	0 / 0
LTR60	629	765	32	32	8	0 / 0	0 / 3	0 / 0
LTR10B1	547	547	121	121	117	0 / 0	0 / 3	2 / 2
MER92C	581	590	56	83	76	1 / 1	0 / 3	6 / 8
LTR19C	779	779	82	91	73	2 / 1	3 / 0	1 / 0
LTR34	701	765	133	166	151	4 / 1	0 / 3	3 / 3
LTR72B	500	546	102	131	131	0 / 2	1 / 2	0 / 1
LTR6B	558	564	135	144	143	2 / 0	0 / 3	2 / 2
MER34D	578	787	163	162	147	7 / 7	0 / 3	2 / 0
MER41C	525	634	538	536	486	11 / 11	3 / 0	7 / 11
PABL_A	660	660	389	392	384	12 / 7	3 / 0	15 / 21
LTR10B	509	633	57	63	57	1 / 1	0 / 3	0 / 0
LTR45	525	604	99	114	112	2 / 6	0 / 3	2 / 1
LTR45B	497	622	309	312	254	6 / 5	0 / 3	3 / 0
MER41E	595	595	245	250	230	13 / 0	0 / 3	4 / 2
MER101	477	570	229	262	237	8 / 2	0 / 3	2 / 2
MER65A	448	531	880	984	964	10 / 9	2 / 1	13 / 12
LTR10A	609	633	249	272	66	4 / 3	0 / 3	2 / 3
LTR24C	634	765	206	212	215	1 / 3	2 / 0	3 / 7
MER34B-int	5,681	9,044	99	75	50	1 / 0	2 / 0	0 / 1
LTR26B	533	622	81	81	79	1 / 2	0 / 2	0 / 0
MER65-int	5,251	9,044	114	144	166	0 / 0	2 / 0	2 / 0
MER41D	556	634	250	281	268	6 / 23	1 / 1	15 / 5
LTR37B	467	468	756	1,201	1,081	16 / 13	0 / 2	9 / 23

Repeat Map

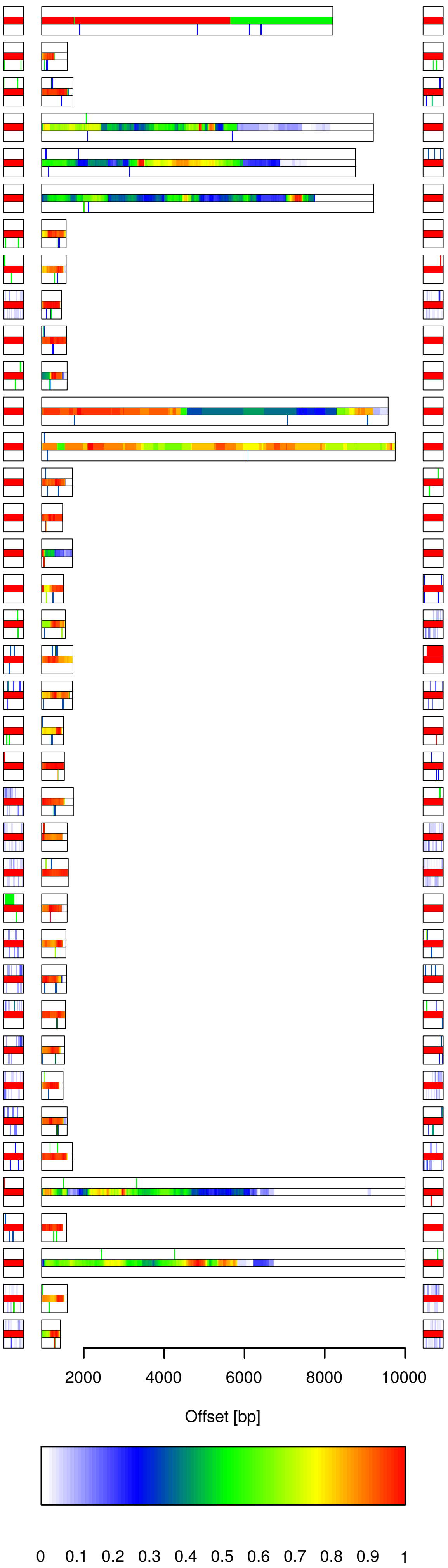

ERV1 (7/8)

Repeat Statistics						Quadruplex Count		
Name	Avg.len.	Max.len.	F5 cov.	Max.cov.	F3 cov.	F5 (+/-)	Rep. (+/-)	F3 (+/-)
ERV1_LTRb	511	511	9	10	9	0 / 0	0 / 2	0 / 0
LTR43-int	5,193	8,436	10	17	17	0 / 0	1 / 1	0 / 0
MER57E3	486	487	86	112	119	1 / 0	2 / 0	7 / 34
LTR38C	709	711	78	79	77	1 / 1	0 / 2	2 / 2
MER57C2	435	542	315	362	333	4 / 4	1 / 1	3 / 3
LTR58	679	679	40	45	44	0 / 1	0 / 2	0 / 0
MER101B	561	597	185	185	105	0 / 0	1 / 1	0 / 0
MER72B	766	768	104	104	99	1 / 2	0 / 2	0 / 1
LTR68	740	740	180	185	99	0 / 1	0 / 2	0 / 1
PrimLTR79	505	622	66	93	91	0 / 0	1 / 1	2 / 5
LTR6A	564	564	219	249	214	3 / 9	0 / 2	2 / 2
MER66D	495	634	120	178	147	3 / 1	0 / 2	2 / 2
MER66A	480	555	238	267	249	1 / 5	1 / 1	9 / 3
ERV-Fc2_LTR	374	374	2	3	3	0 / 0	0 / 2	0 / 0
PABL_B	667	667	222	260	256	5 / 4	0 / 2	11 / 8
LTR54B	499	510	578	587	516	6 / 4	0 / 2	4 / 9
LTR4	596	596	75	88	88	3 / 1	0 / 2	1 / 0
MER110-int	4,744	8,270	32	90	76	0 / 0	1 / 1	0 / 1
LTR1C3	638	638	57	57	36	0 / 1	0 / 2	0 / 2
MER101-int	6,327	9,044	55	187	92	1 / 0	2 / 0	0 / 0
LTR65	669	669	210	212	211	5 / 3	0 / 2	2 / 5
V24B_Prim-int	7,527	8,270	46	49	41	0 / 1	2 / 0	1 / 0
LTR51	679	923	248	274	268	3 / 0	1 / 1	3 / 2
LTR9	614	779	572	613	562	2 / 5	0 / 2	3 / 3
LTR61	560	675	104	125	126	1 / 5	0 / 2	6 / 4
MER34C	554	707	45	109	69	0 / 0	0 / 2	0 / 0
LTR54	509	510	624	683	683	16 / 14	2 / 0	7 / 11
LTR24	473	765	200	219	219	3 / 3	0 / 2	0 / 9
MER57E2	402	402	59	60	60	1 / 1	0 / 1	0 / 2
LTR43B	594	885	74	146	141	0 / 0	1 / 0	1 / 0
LTR71A	459	459	144	146	143	6 / 2	1 / 0	2 / 2
MER57A1	432	435	857	882	864	5 / 15	1 / 0	6 / 7
LTR24B	589	765	223	272	275	6 / 4	0 / 1	4 / 8
MER57B1	401	434	754	849	756	5 / 10	0 / 1	7 / 7
LTR38-int	2,967	8,120	35	34	4	0 / 0	0 / 1	0 / 0
MER65B	519	531	131	129	82	2 / 4	0 / 1	0 / 1
MER4A1_	601	900	320	325	324	3 / 2	0 / 1	12 / 4
MER65D	475	531	249	272	238	3 / 4	0 / 1	2 / 0

Repeat Map

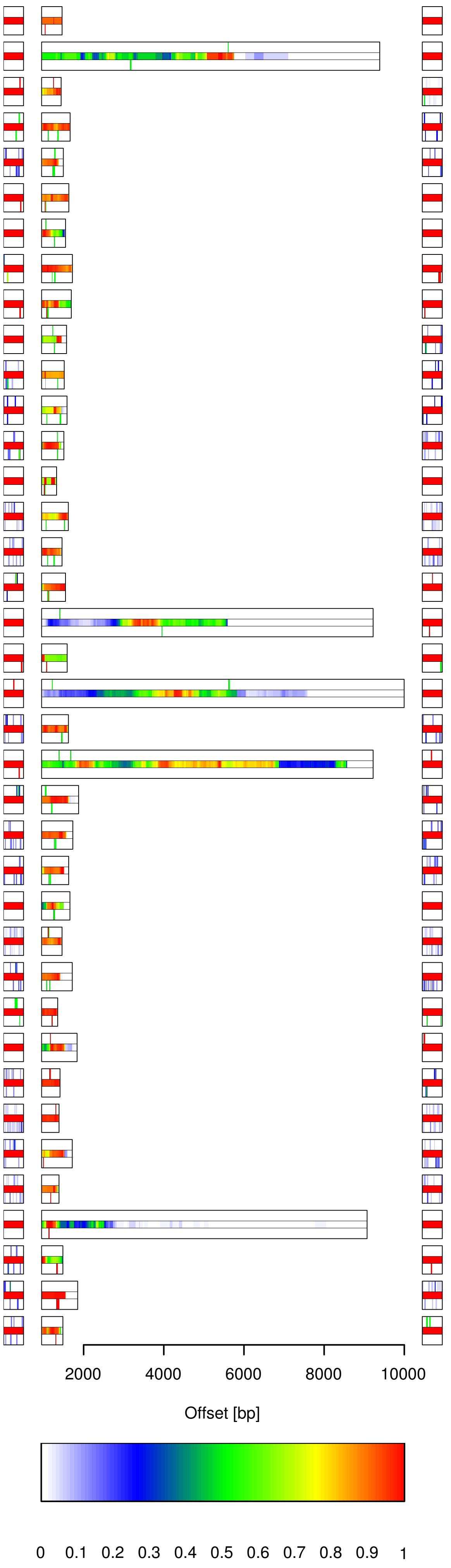

ERV1 (8/8)

Name	Avg.len.	Max.len.	F5 cov.	Max.cov.	F3 cov.	F5 (+/-)	Rep. (+/-)	F3 (+/-)
	**Repeat Statistics**					**Quadruplex Count**		
IERV1_LTRc	491	491	29	30	28	0 / 1	0 / 1	1 / 0
LTR73	572	572	152	156	154	1 / 2	1 / 0	4 / 7
MER83C	373	378	104	134	131	2 / 4	0 / 1	1 / 1
LTR06	476	476	53	74	73	0 / 0	0 / 1	1 / 1
MER57E1	374	435	366	389	371	3 / 3	0 / 1	6 / 5
MER50C	777	782	42	57	48	0 / 0	1 / 0	0 / 0
LTR39-int	8,285	9,044	14	24	24	0 / 0	0 / 1	0 / 0
MER57F	434	487	282	288	277	1 / 4	0 / 1	5 / 2
RV-Fc1_LTR2	425	425	3	3	3	0 / 0	0 / 0	0 / 0
ERV1_LTRe	537	537	3	29	26	0 / 0	0 / 0	0 / 0
IERV-Fc1-int	4,629	4,629	5	7	6	0 / 0	0 / 0	0 / 0
LTR109A2	425	431	15	21	17	0 / 0	0 / 0	0 / 0
MER92A	419	636	91	101	100	1 / 2	0 / 0	3 / 3
MER95	431	431	100	100	97	0 / 1	0 / 0	0 / 1
PRIMAX-int	4,691	7,613	5	20	2	0 / 0	0 / 0	0 / 0
MER57D	413	542	300	362	351	1 / 4	0 / 0	4 / 4
ERV1_LTRd	535	535	13	15	14	0 / 1	0 / 0	1 / 0
LTR76	674	675	116	161	148	4 / 2	0 / 0	2 / 0
MER51D	657	657	45	113	81	0 / 0	0 / 0	0 / 2
LTR44	519	519	133	140	130	12 / 1	0 / 0	4 / 3
MER57C1	395	542	125	141	127	0 / 1	0 / 0	2 / 4
LTR2	463	501	759	787	779	12 / 8	0 / 0	12 / 7
MER84	507	507	249	248	194	1 / 3	0 / 0	2 / 0
LTR23-int	5,454	9,044	3	34	38	0 / 0	0 / 0	0 / 1
LTR10C	592	633	379	428	406	8 / 9	0 / 0	8 / 6
LTR7A	451	472	8	12	12	1 / 0	0 / 0	0 / 0
LTR10E	632	633	189	208	204	1 / 2	0 / 0	1 / 4
LTR2B	483	501	302	304	305	4 / 5	0 / 0	0 / 5
LTR64	528	528	211	219	217	8 / 4	0 / 0	7 / 4

Repeat Map

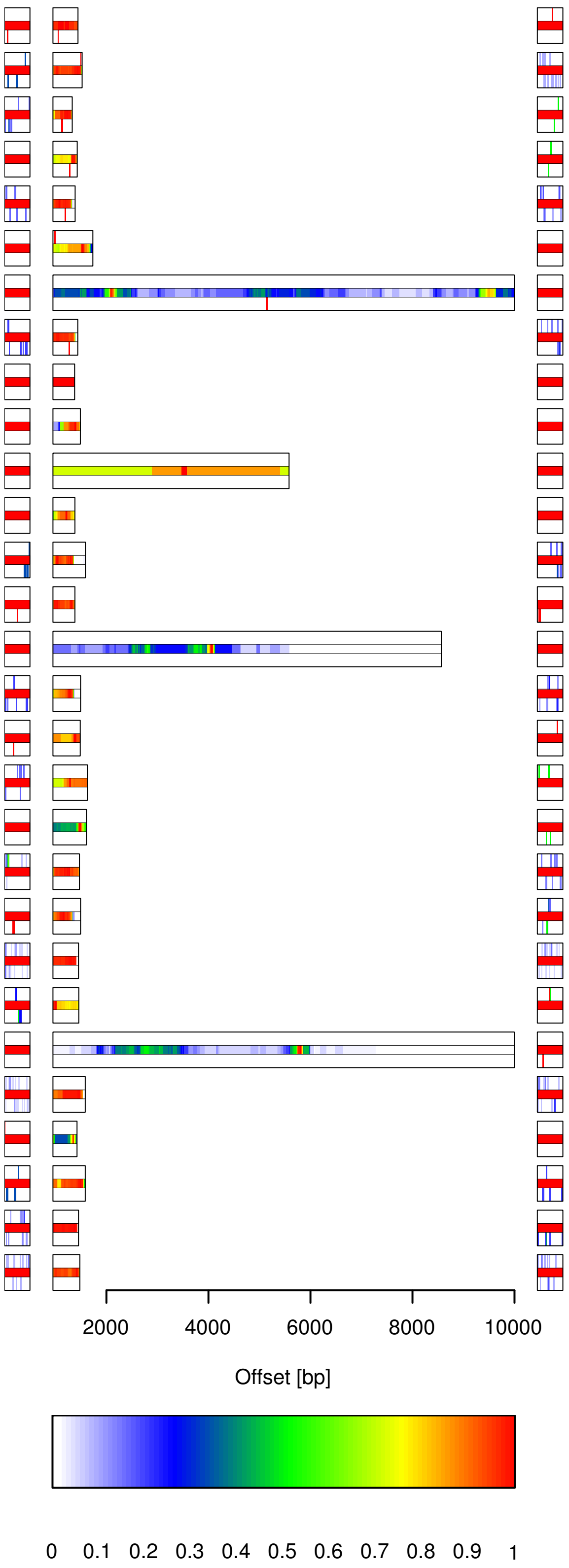

hAT-Charlie (1/2)

Repeat Statistics						Quadruplex Count		
Name	Avg.len.	Max.len.	F5 cov.	Max.cov.	F3 cov.	F5 (+/-)	Rep. (+/-)	F3 (+/-)
MER1B	341	2,706	4,335	4,731	4,675	64 / 47	9 / 622	40 / 46
MER20	220	783	12,075	14,848	13,756	258 / 281	200 / 205	291 / 370
MER1A	528	529	2,593	2,760	2,710	49 / 25	8 / 249	42 / 40
MER5A	189	189	18,066	28,451	20,815	371 / 410	113 / 129	465 / 486
MER5B	178	189	14,947	19,121	14,288	362 / 329	107 / 131	286 / 286
Charlie15a	224	1,061	1,039	2,894	1,942	29 / 29	110 / 23	59 / 47
MER5A1	166	189	10,637	13,890	11,189	248 / 221	6 / 86	207 / 210
MER102b	396	2,428	2,255	3,070	1,579	71 / 78	7 / 70	28 / 44
MER102c	395	2,428	730	2,712	2,291	27 / 15	3 / 73	43 / 67
Charlie8	2,152	2,428	1,891	1,849	405	77 / 73	5 / 50	8 / 10
MER102a	369	2,428	871	2,055	1,414	34 / 27	9 / 45	32 / 31
MER20B	741	783	1,797	2,031	1,519	30 / 41	47 / 5	32 / 35
Charlie4z	167	1,961	2,390	5,235	3,339	71 / 63	38 / 3	99 / 94
MER117	197	197	2,717	3,821	1,956	49 / 56	33 / 0	34 / 49
Charlie1a	1,454	1,455	1,681	1,918	2,036	18 / 25	13 / 15	24 / 37
Charlie17a	221	1,204	549	1,028	878	10 / 6	23 / 4	9 / 16
Charlie3	2,443	2,706	178	173	175	2 / 2	0 / 21	0 / 3
MER30	231	2,873	3,265	3,523	3,463	56 / 57	0 / 18	68 / 57
MER103C	297	1,166	2,386	7,365	3,068	50 / 57	13 / 4	66 / 61
Charlie16a	342	342	346	1,460	630	9 / 13	9 / 7	11 / 15
Charlie2a	2,860	2,861	786	646	186	16 / 12	3 / 12	2 / 1
Charlie2b	2,782	2,861	567	1,033	1,285	8 / 4	8 / 6	34 / 26
Charlie17b	1,202	1,204	149	181	129	3 / 1	9 / 3	1 / 3
MER58A	225	2,420	9,642	12,171	10,633	164 / 143	3 / 9	152 / 158
MER58B	341	2,420	3,461	5,023	4,856	67 / 58	4 / 6	78 / 96
Charlie1b	526	2,781	1,292	1,517	1,568	15 / 29	7 / 2	35 / 33
Charlie7	2,591	2,612	1,135	888	733	28 / 15	4 / 4	11 / 14
MER113	521	521	946	2,083	1,537	38 / 19	6 / 2	57 / 70
Charlie20a	807	807	239	355	204	4 / 6	6 / 2	5 / 10
MER106B	1,015	1,059	263	328	380	6 / 5	7 / 0	3 / 12
MER58C	217	2,420	1,407	1,975	1,847	17 / 19	5 / 2	31 / 34
Charlie25	2,524	2,524	214	194	186	1 / 4	1 / 5	9 / 0
Charlie1	2,775	2,781	723	642	531	18 / 13	3 / 3	3 / 13
Charlie19a	386	386	447	1,018	564	12 / 18	6 / 0	25 / 19
MER106A	236	236	525	635	519	12 / 5	5 / 0	12 / 5
Charlie4a	509	1,961	1,553	1,915	1,718	46 / 38	5 / 0	74 / 63
MER3	211	2,624	7,324	8,651	6,763	162 / 136	3 / 2	112 / 126
MER33	323	324	5,599	6,285	5,635	123 / 131	4 / 0	117 / 140

Repeat Map

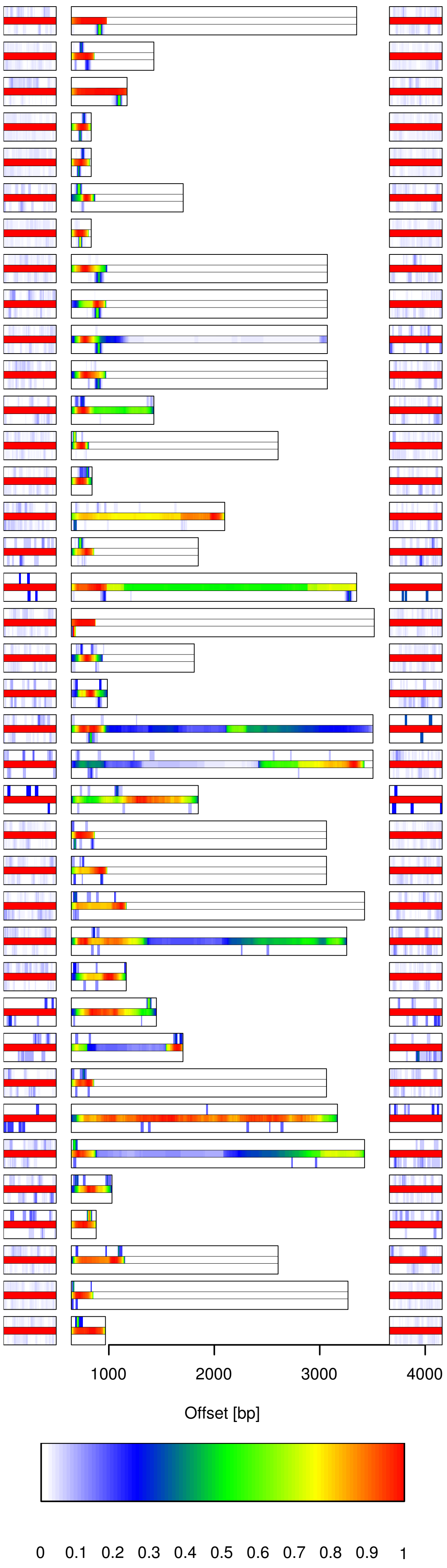

hAT-Charlie (2/2)

Repeat Statistics						Quadruplex Count		
Name	Avg.len.	Max.len.	F5 cov.	Max.cov.	F3 cov.	F5 (+/-)	Rep. (+/-)	F3 (+/-)
Charlie13a	1,512	1,514	132	159	106	0 / 0	3 / 1	2 / 1
Charlie23a	339	339	396	1,048	512	7 / 4	4 / 0	7 / 9
Chap1_Mam	342	342	373	1,167	286	7 / 4	2 / 2	4 / 1
Charlie21a	1,213	1,213	167	239	162	1 / 6	2 / 1	6 / 3
Charlie29a	719	719	509	894	744	12 / 11	2 / 1	8 / 22
MER113A	314	521	698	1,046	699	19 / 16	3 / 0	31 / 32
Charlie17	2,908	2,916	79	245	282	0 / 2	1 / 1	5 / 8
MER119	583	583	590	641	637	6 / 6	0 / 2	9 / 11
Charlie22a	491	491	315	563	459	9 / 9	0 / 2	8 / 12
MER30B	192	230	139	186	182	3 / 2	0 / 1	2 / 0
Charlie26a	325	325	102	352	244	1 / 0	0 / 1	8 / 7
Charlie10	2,724	2,822	535	491	280	8 / 3	1 / 0	6 / 5
Charlie7a	298	2,612	574	1,090	757	8 / 7	1 / 0	14 / 17
MER105	203	203	438	609	449	6 / 4	0 / 1	6 / 3
MER58D	385	386	299	786	798	5 / 3	0 / 1	9 / 22
Charlie15b	1,059	1,061	412	598	710	5 / 6	1 / 0	15 / 18
MER112	272	2,757	2,332	3,197	2,197	48 / 48	1 / 0	40 / 33
Charlie9	2,507	2,757	551	525	406	18 / 13	1 / 0	11 / 16
Charlie5	2,579	2,624	690	1,048	1,166	18 / 13	0 / 1	22 / 33
harlie1b_Mars	1,762	1,762	1	1	0	0 / 0	0 / 0	0 / 0
eshire_Mars_	1,393	1,393	2	3	2	0 / 0	0 / 0	0 / 0
Charlie12	2,873	2,873	4	3	2	0 / 0	0 / 0	0 / 0
Charlie6	3,500	3,500	47	48	47	2 / 0	0 / 0	1 / 5
Charlie11	2,196	2,196	19	28	22	0 / 0	0 / 0	0 / 1
Charlie4	1,873	2,450	40	48	48	1 / 0	0 / 0	1 / 3
Charlie10b	242	280	184	218	136	2 / 0	0 / 0	3 / 0
Charlie14a	1,148	1,166	190	143	130	4 / 3	0 / 0	0 / 4
MER113B	303	314	158	422	226	3 / 2	0 / 0	8 / 0
Charlie13b	512	512	70	286	124	2 / 2	0 / 0	0 / 0
MER5C1	281	2,822	352	457	370	6 / 6	0 / 0	7 / 10
Charlie10a	280	280	86	231	128	0 / 1	0 / 0	1 / 4
Charlie24	2,450	2,450	101	702	764	1 / 0	0 / 0	28 / 18
Cheshire	2,378	2,420	354	256	252	2 / 3	0 / 0	2 / 2
Charlie18a	342	342	719	1,637	1,028	18 / 12	0 / 0	24 / 37
MER5C	316	2,822	694	960	738	8 / 9	0 / 0	25 / 18
MER107	200	200	293	296	220	5 / 8	0 / 0	2 / 1

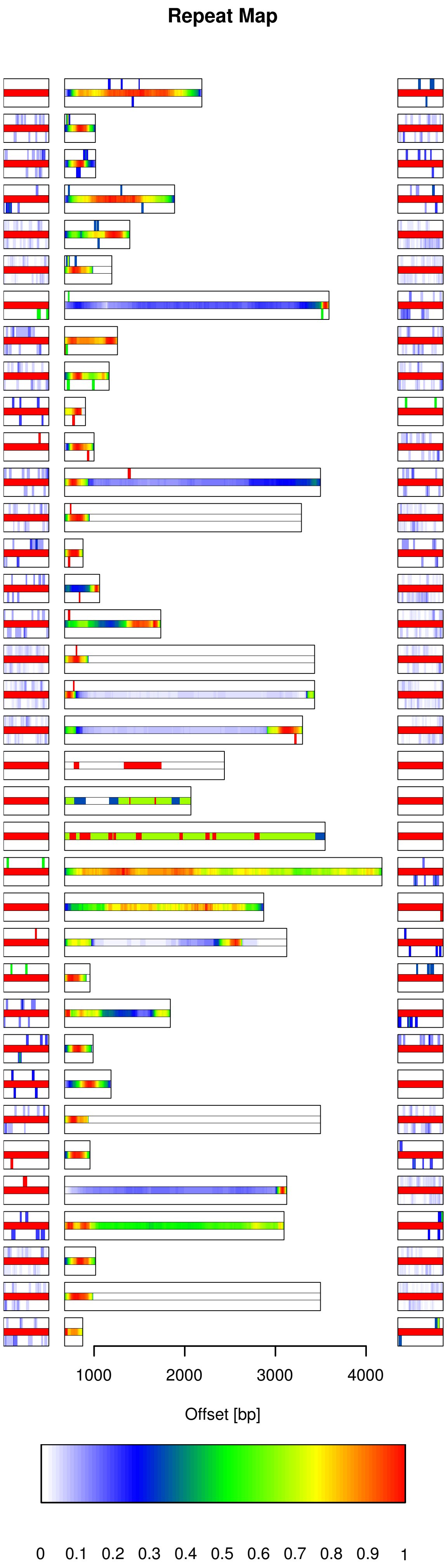

L1 (1/4)

Repeat Statistics						Quadruplex Count		
Name	Avg.len.	Max.len.	F5 cov.	Max.cov.	F3 cov.	F5 (+/-)	Rep. (+/-)	F3 (+/-)
L1PA4	6,159	8,042	1,629	9,736	10,382	12 / 6	7,677 / 6	84 / 76
L1PA3	6,164	9,304	1,715	8,642	9,050	14 / 12	7,333 / 48	74 / 46
L1PA5	6,163	8,155	1,321	9,252	9,799	14 / 3	6,849 / 10	88 / 76
L1PA7	6,226	10,144	1,700	9,636	10,161	6 / 8	6,747 / 35	65 / 61
L1PB1	6,407	10,020	1,188	6,775	7,734	7 / 1	3,920 / 89	81 / 57
L1PA2	6,157	8,150	1,114	4,010	4,297	5 / 7	3,445 / 26	23 / 24
L1PA8	6,205	8,167	435	6,174	6,810	1 / 1	3,446 / 15	75 / 65
L1PA6	6,162	7,882	1,246	4,691	4,885	8 / 5	3,126 / 9	52 / 43
L1PA16	6,276	8,161	482	8,545	9,451	3 / 1	1,602 / 105	88 / 71
L1PA10	6,248	8,310	683	4,582	4,951	1 / 2	1,652 / 29	50 / 58
L1MB3	6,216	8,042	258	9,215	10,876	0 / 1	1,495 / 18	312 / 240
L1MB7	6,301	9,304	604	10,001	12,115	7 / 8	1,220 / 47	285 / 242
L1MA2	6,807	9,461	637	3,878	4,359	6 / 1	1,116 / 40	41 / 50
L1PA13	6,338	8,160	642	5,602	6,079	3 / 1	935 / 169	47 / 42
L1MA3	6,826	9,461	737	4,285	4,967	5 / 3	961 / 43	38 / 34
L1ME1	6,256	9,304	1,344	11,632	14,345	16 / 3	925 / 35	228 / 194
L1MB8	6,274	9,304	327	7,774	9,333	4 / 1	933 / 16	195 / 149
L1HS	6,145	6,633	448	1,015	1,081	1 / 5	939 / 8	6 / 12
L1PB4	6,240	8,161	134	4,757	5,355	0 / 0	857 / 32	46 / 54
L1PA8A	6,213	8,160	280	1,443	1,694	3 / 1	863 / 5	14 / 11
L1MA9	6,436	8,745	246	7,741	9,908	1 / 0	780 / 52	100 / 84
L1PA11	6,338	9,304	217	2,884	3,071	0 / 0	741 / 10	26 / 30
L1MB5	6,280	8,386	195	4,602	5,649	2 / 2	718 / 12	183 / 174
L1MC1	6,355	9,304	561	6,203	7,472	3 / 2	704 / 9	83 / 104
L1PB2	6,281	7,583	117	1,740	1,918	0 / 0	695 / 12	29 / 18
L1PA15	6,368	9,304	377	5,034	5,448	1 / 4	569 / 117	46 / 42
HAL1	2,505	2,991	3,838	4,330	5,030	49 / 38	432 / 216	85 / 67
L1MA1	6,730	9,140	353	2,391	2,757	1 / 0	620 / 27	19 / 25
L1MA8	6,436	9,414	150	5,815	7,192	0 / 0	547 / 34	71 / 56
L1PREC2	6,419	8,300	304	2,974	3,536	0 / 0	431 / 120	25 / 20
L1MB2	6,261	8,438	95	4,318	5,068	0 / 1	531 / 7	84 / 75
L1MD2	6,418	8,580	729	3,566	5,168	18 / 18	487 / 32	84 / 70
L1MA4	6,517	9,461	154	5,185	6,362	1 / 0	484 / 27	68 / 59
L1PA17	6,324	8,441	264	2,951	3,229	1 / 1	478 / 29	31 / 34
L1MA7	6,397	7,995	83	4,726	5,800	0 / 0	486 / 14	60 / 66
L1MC2	6,390	9,477	196	2,761	3,461	1 / 1	485 / 4	45 / 34
L1ME4b	1,331	9,304	2,552	7,324	7,324	29 / 34	461 / 9	176 / 163
L1PB3	6,311	8,501	145	2,078	2,277	1 / 1	443 / 14	32 / 19

Repeat Map

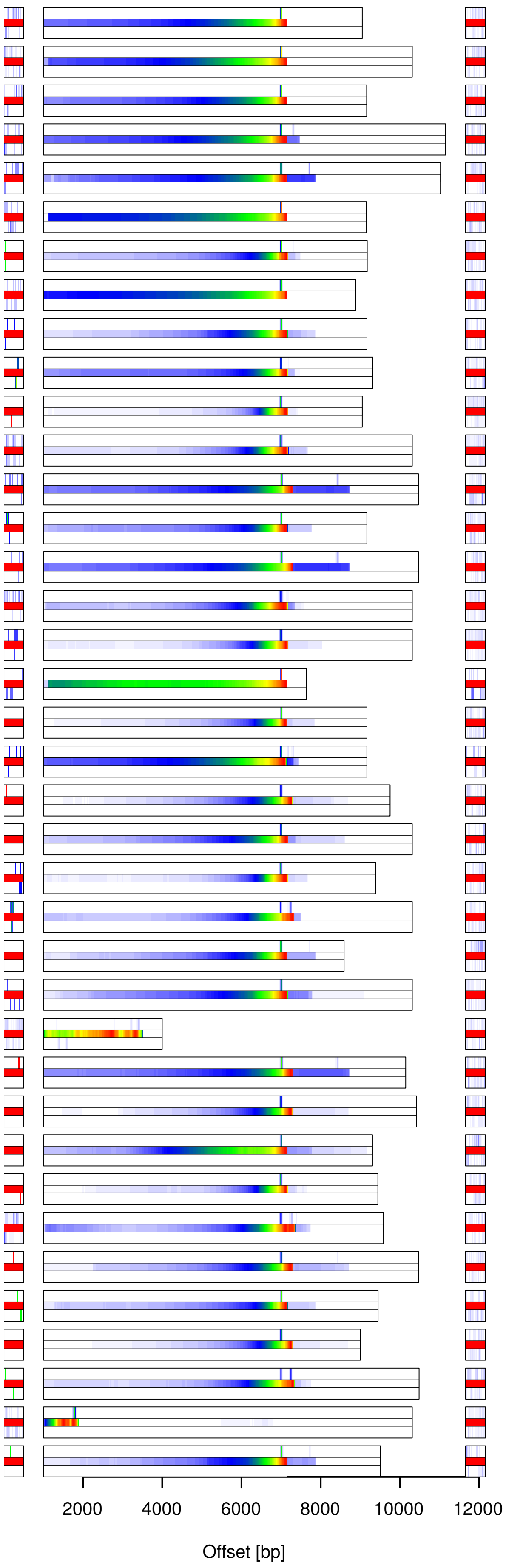

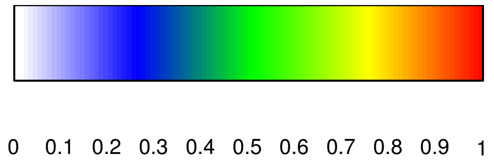

Repeat Statistics						Quadruplex Count		
Name	Avg.len.	Max.len.	F5 cov.	Max.cov.	F3 cov.	F5 (+/-)	Rep. (+/-)	F3 (+/-)
L1MA4A	6,578	8,697	454	2,799	3,276	1 / 3	369 / 77	32 / 19
L1MB4	6,303	9,304	503	4,221	4,956	7 / 2	392 / 28	80 / 66
L1PA14	6,486	8,163	176	1,795	1,933	0 / 1	357 / 26	13 / 15
L1ME3A	6,224	9,304	423	7,057	8,764	3 / 2	332 / 12	140 / 142
L1MD1	6,402	8,433	468	2,201	2,668	4 / 5	315 / 26	42 / 29
L1MC3	7,588	9,304	263	4,310	5,962	1 / 1	255 / 59	77 / 90
L1MA6	6,512	8,118	92	2,686	3,393	0 / 0	293 / 15	29 / 34
L1MA5A	6,385	8,292	77	2,068	2,322	0 / 0	282 / 22	13 / 16
L1MA5	6,499	7,995	156	2,280	2,718	0 / 0	252 / 18	25 / 26
L1MC5	9,067	10,612	75	5,481	8,297	0 / 1	249 / 7	151 / 166
L1MB1	6,296	8,042	87	2,765	3,601	2 / 2	238 / 9	45 / 33
L1M1	7,250	9,414	1,912	1,723	485	6 / 11	123 / 119	4 / 5
L1MC4	7,735	9,802	616	7,044	11,168	5 / 3	179 / 36	190 / 180
L1ME2	6,243	9,304	432	4,599	6,015	1 / 7	203 / 9	92 / 102
L1ME3	6,219	8,790	207	3,840	4,955	4 / 0	197 / 8	106 / 96
L1PA12	6,568	8,169	252	812	953	3 / 1	166 / 17	6 / 4
L1ME3B	6,252	8,411	212	2,779	3,970	0 / 1	165 / 1	82 / 60
L1ME3Cz	2,019	9,304	1,440	3,397	3,863	19 / 10	156 / 9	86 / 58
L1MC5a	2,952	9,372	1,060	4,513	6,418	11 / 27	141 / 13	179 / 100
L1ME2z	6,398	9,304	148	2,565	4,152	0 / 0	150 / 2	72 / 59
L1MC4a	7,672	9,304	86	2,822	3,902	0 / 0	146 / 6	65 / 55
L1ME3G	1,746	9,304	2,699	5,665	2,348	32 / 21	142 / 8	46 / 48
L1M2	6,468	8,438	619	1,356	856	3 / 0	26 / 122	16 / 6
L1MA10	6,361	8,438	34	3,325	3,635	0 / 0	130 / 8	60 / 34
L1M4b	6,867	7,963	1,030	771	80	21 / 25	36 / 91	1 / 5
HAL1ME	2,467	2,507	1,178	1,627	2,404	10 / 13	90 / 31	47 / 31
L1MDa	6,595	9,303	1,723	1,334	70	14 / 16	52 / 66	2 / 2
L1MEc	6,384	8,237	5,208	4,065	200	43 / 37	83 / 33	4 / 1
L1MEd	6,713	8,522	4,928	3,432	285	61 / 50	94 / 19	7 / 2
L1PBa	6,853	7,566	758	688	97	2 / 0	51 / 45	0 / 0
L1MD3	7,811	9,414	60	2,367	3,151	0 / 0	88 / 8	65 / 41
L1ME5	6,195	7,103	16	1,527	2,356	0 / 0	86 / 5	46 / 42
L1M4	6,483	9,014	1,586	2,660	1,821	10 / 7	39 / 52	36 / 31
L1MCa	6,517	8,203	1,833	1,402	40	19 / 16	64 / 26	0 / 0
L1MEf	6,317	8,580	2,217	2,098	147	21 / 19	55 / 30	0 / 2
L1ME3E	6,277	8,042	148	1,560	2,302	2 / 0	79 / 5	36 / 39
L1PA15-16	6,906	8,161	319	327	55	0 / 3	22 / 59	0 / 1
L1M5	6,160	8,042	1	8,303	12,541	0 / 0	56 / 25	232 / 212

Repeat Map

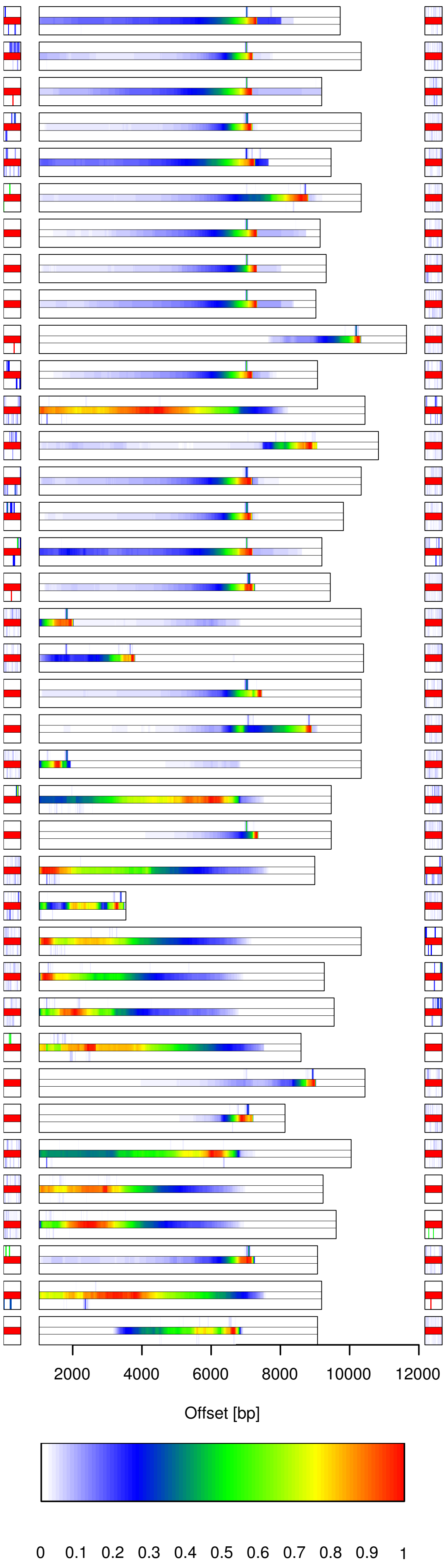

Repeat Statistics						Quadruplex Count		
Name	Avg.len.	Max.len.	F5 cov.	Max.cov.	F3 cov.	F5 (+/-)	Rep. (+/-)	F3 (+/-)
L1ME3F	6,267	8,194	61	1,526	2,340	0 / 0	75 / 2	58 / 36
L1ME3C	6,118	8,902	63	1,439	2,061	0 / 1	64 / 1	33 / 26
L1M3e	7,195	9,204	291	193	12	6 / 2	2 / 58	0 / 0
L1ME3D	6,272	8,413	122	1,399	2,091	2 / 0	58 / 1	26 / 34
L1M4c	6,528	8,438	638	665	70	7 / 3	13 / 46	0 / 0
L1MCc	6,646	8,790	1,120	899	24	10 / 6	27 / 27	0 / 1
L1ME4a	6,120	9,304	219	16,511	24,547	4 / 0	42 / 12	514 / 513
L1P4a	6,676	8,161	154	163	22	1 / 1	14 / 39	0 / 0
HAL1b	2,423	6,633	393	1,581	1,940	3 / 7	39 / 11	47 / 39
L1MEg	6,313	9,098	3,443	2,014	150	28 / 22	41 / 9	1 / 5
L1MEh	2,633	7,357	490	619	187	11 / 4	24 / 15	2 / 2
L1MCb	6,403	8,790	879	642	8	11 / 3	19 / 17	0 / 0
L1MDb	6,505	7,661	351	234	7	6 / 4	6 / 29	0 / 0
L1MEi	2,965	7,565	892	964	629	6 / 16	26 / 8	5 / 1
L1PBa1	6,783	8,161	249	207	18	1 / 1	26 / 7	1 / 0
L1MEj	2,507	7,517	216	495	304	2 / 2	26 / 5	1 / 2
L1M2a	7,259	7,719	162	118	6	0 / 0	2 / 27	0 / 0
L1P3	6,225	8,310	500	694	354	3 / 10	22 / 7	1 / 2
L1M2c	6,951	7,752	128	111	4	1 / 1	2 / 26	0 / 0
L1M7	5,879	7,494	848	628	97	10 / 7	21 / 5	2 / 1
L1M6	6,327	8,136	1,264	1,036	114	9 / 13	19 / 4	4 / 5
HAL1M8	2,987	2,991	481	411	403	6 / 4	17 / 6	6 / 5
L1M4a2	3,388	7,605	429	402	221	3 / 5	12 / 10	0 / 0
L1M8	2,716	7,357	401	335	174	4 / 4	18 / 4	0 / 2
L1M2b	7,214	7,508	107	92	4	0 / 3	3 / 17	0 / 0
L1M4a1	3,385	7,550	479	526	170	3 / 5	10 / 10	3 / 1
L1MEb	6,097	8,055	858	598	7	19 / 6	15 / 4	0 / 0
L1P4d	6,944	7,228	72	61	4	1 / 0	2 / 15	0 / 0
L1M3c	6,742	8,040	243	175	13	1 / 5	3 / 14	0 / 0
L1P2	6,201	8,179	504	635	289	2 / 5	13 / 3	3 / 4
L1P4b	7,454	8,441	79	66	4	0 / 0	2 / 13	0 / 0
L1MD	6,189	8,042	0	1,173	420	0 / 0	9 / 4	11 / 8
L1M3	6,163	7,995	0	1,042	1,181	0 / 0	10 / 3	25 / 29
L1M6B	1,087	7,357	294	439	28	2 / 2	7 / 5	1 / 0
L1M3b	6,815	7,731	165	129	3	1 / 0	2 / 9	0 / 0
L1M2a1	7,114	8,142	61	53	0	0 / 1	3 / 7	0 / 0
L1ME4c	1,395	9,304	626	3,589	1,725	7 / 9	5 / 5	37 / 37
L1P4e	6,533	7,601	91	65	1	0 / 0	1 / 8	0 / 0

Repeat Map

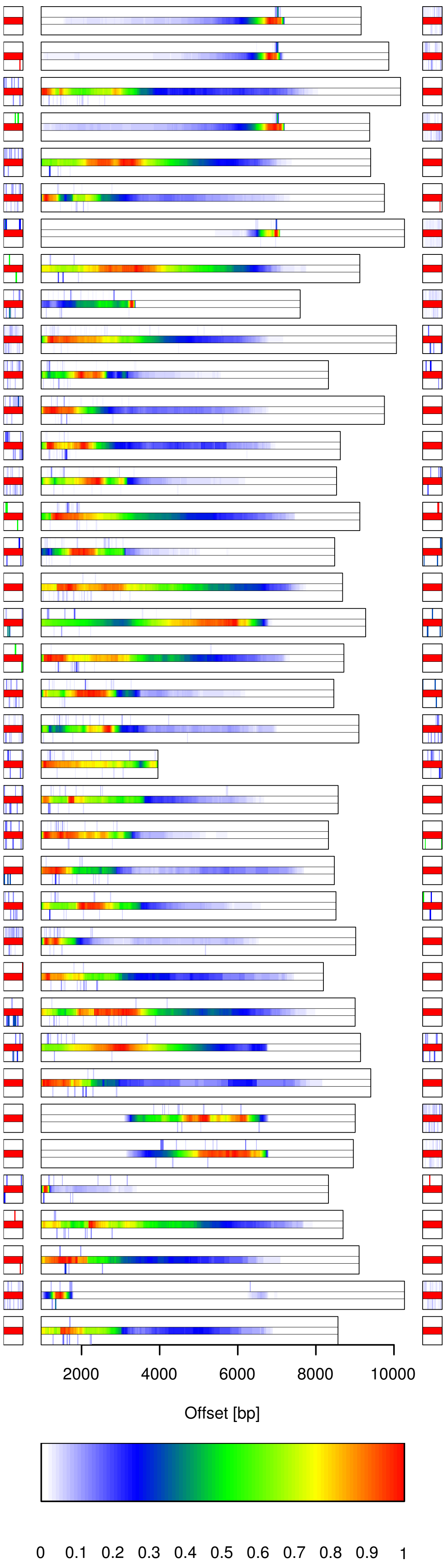

L1 (4/4)

Name	Avg.len.	Max.len.	F5 cov.	Max.cov.	F3 cov.	F5 (+/-)	Rep. (+/-)	F3 (+/-)
	**Repeat Statistics**					**Quadruplex Count**		
L1M3a	6,299	7,731	247	175	6	3 / 1	1 / 7	0 / 0
L1MEg1	6,668	8,194	404	254	8	4 / 3	6 / 2	0 / 0
L1M3f	6,791	8,155	134	152	12	1 / 1	1 / 7	0 / 0
L1P1	6,151	7,995	448	741	243	4 / 4	4 / 3	3 / 1
L1MEa	6,039	7,086	173	117	3	0 / 2	4 / 2	0 / 0
L1MC	6,447	8,042	0	1,066	381	0 / 0	3 / 3	5 / 7
L1P3b	6,094	6,995	58	47	0	0 / 0	2 / 3	0 / 0
L1P4	6,156	7,995	0	795	769	0 / 0	3 / 2	8 / 5
L1M3d	6,318	8,438	208	140	4	1 / 1	1 / 3	0 / 0
L1P4c	6,755	8,155	25	17	0	0 / 0	0 / 3	0 / 0
L1MEg2	6,732	7,652	342	249	7	2 / 1	1 / 2	0 / 0
L1PB	6,151	7,995	2	446	194	0 / 0	1 / 1	2 / 0
L1PBb	6,717	7,168	101	70	5	0 / 0	0 / 1	0 / 0
L1M3de	6,806	8,438	131	101	5	0 / 2	1 / 0	0 / 0
L1P5	6,161	6,867	0	253	481	0 / 0	1 / 0	2 / 3
L1P	6,174	7,995	0	105	0	0 / 0	0 / 0	0 / 0
L1M	6,283	7,995	0	536	23	0 / 0	0 / 0	0 / 0

Repeat Map

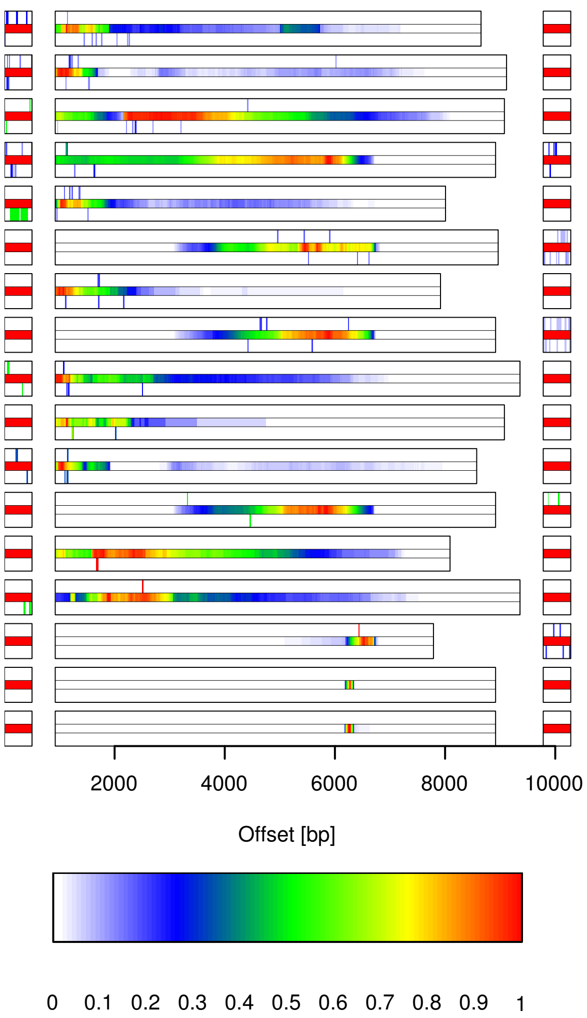

L2 (1/1)

	Repeat Statistics					Quadruplex Count		
Name	Avg.len.	Max.len.	F5 cov.	Max.cov.	F3 cov.	F5 (+/-)	Rep. (+/-)	F3 (+/-)
L2a	3,423	3,426	627	101,521	119,962	13 / 12	112 / 5,456	3,171 / 2,861
L2b	3,385	3,426	84	60,697	76,447	3 / 2	154 / 2,920	3,292 / 3,041
L2c	3,392	3,426	107	80,970	108,477	3 / 0	98 / 2,797	2,910 / 2,646
L2	3,419	3,419	1,543	15,280	1,179	30 / 25	20 / 1,744	22 / 27

Repeat Map

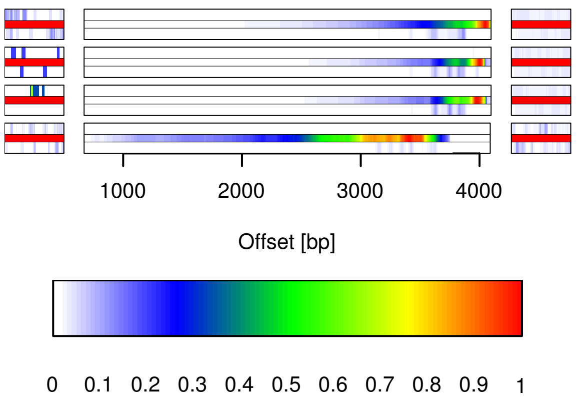

MIR (1/1)

Name	Avg.len.	Max.len.	F5 cov.	Max.cov.	F3 cov.	F5 (+/-)	Rep. (+/-)	F3 (+/-)
MIRb	267	268	85,757	181,333	98,089	3,166 / 2,855	772 / 265	2,869 / 3,222
MIR3	212	268	24,092	77,567	35,884	1,111 / 1,126	459 / 304	1,212 / 1,305
MIRc	266	268	37,478	80,871	29,456	1,350 / 1,273	323 / 220	728 / 764
MIR	262	268	79,465	139,351	93,928	2,466 / 2,353	283 / 169	2,264 / 2,392

Table column groups: Repeat Statistics (Name through F3 cov.); Quadruplex Count (F5 (+/-) through F3 (+/-)).

Repeat Map

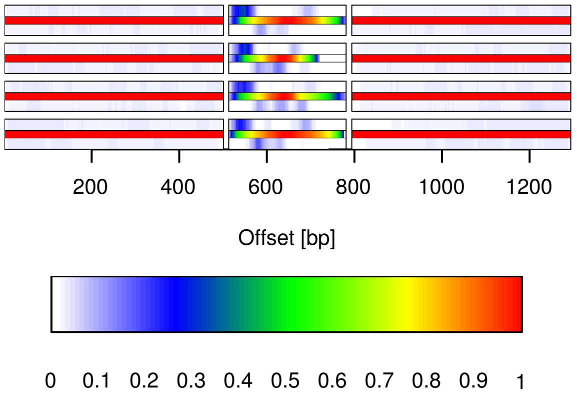

SVA (1/1)

Repeat Statistics						Quadruplex Count		
Name	Avg.len.	Max.len.	F5 cov.	Max.cov.	F3 cov.	F5 (+/-)	Rep. (+/-)	F3 (+/-)
SVA_D	1,386	1,386	623	888	809	8 / 62	969 / 517	12 / 11
SVA_F	1,375	1,375	100	638	269	1 / 13	1,220 / 80	6 / 3
SVA_B	1,383	1,383	326	421	422	1 / 47	11 / 218	7 / 4
SVA_C	1,384	1,384	205	263	254	1 / 16	31 / 176	9 / 2
SVA_E	1,382	1,382	56	117	103	0 / 24	183 / 16	4 / 4
SVA_A	1,387	1,387	86	148	142	1 / 8	6 / 45	3 / 2

Repeat Map

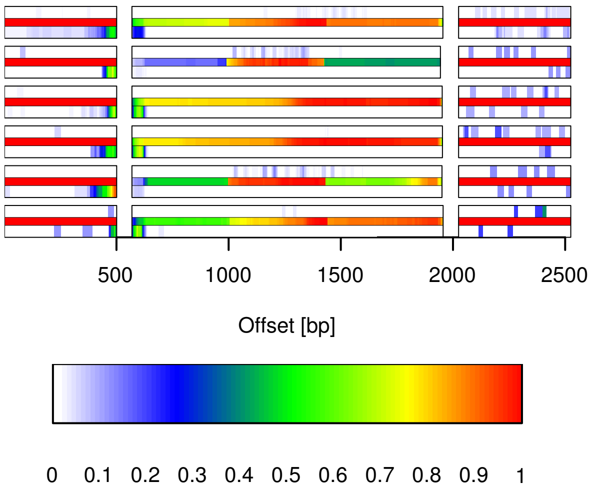

Supplement: Supplementary file 1 — Additional file 1: A detailed visualization of PQS coverage of main human transposable element families and subfamilies. (PDF 4 MB) [file 12864_2014_6983_MOESM1_ESM.pdf]
